# LCAT1 is an oncogenic LncRNA by stabilizing the IGF2BP2-CDC6 axis

**DOI:** 10.1038/s41419-022-05316-4

**Published:** 2022-10-18

**Authors:** Juze Yang, Xinyi Qian, Qiongzi Qiu, Lingling Xu, Meidie Pan, Jia Li, Jiayi Ren, Bingjian Lu, Ting Qiu, Enguo Chen, Kejing Ying, Honghe Zhang, Yan Lu, Pengyuan Liu

**Affiliations:** 1 Department of Respiratory Medicine, Sir Run Run Shaw Hospital and Institute of Translational Medicine, Zhejiang University School of Medicine, Hangzhou, Zhejiang 310016 China; 2 Zhejiang Provincial Key Laboratory of Precision Diagnosis and Therapy for Major Gynecological Diseases, Center for Uterine Cancer Diagnosis & Therapy Research of Zhejiang Province, Department of Gynecologic Oncology, Women’s Hospital and Institute of Translational Medicine, Zhejiang University School of Medicine, Hangzhou, Zhejiang 310006 China; 3 Department of Pathology, Research Unit of Intelligence Classification of Tumor Pathology and Precision Therapy, Chinese Academy of Medical Sciences, Zhejiang University School of Medicine, Hangzhou, 310058 Zhejiang China; 4 Cancer center, Zhejiang University, Hangzhou, Zhejiang 310013 China; 5 Department of Physiology and Center of Systems Molecular Medicine, Medical College of Wisconsin, Milwaukee, WI USA

**Keywords:** Long non-coding RNAs, Oncogenes

## Abstract

Long non-coding RNAs (lncRNAs) is known to play vital roles in modulating tumorigenesis. We previously reported that LCAT1, a novel lncRNA, promotes the growth and metastasis of lung cancer cells both in vitro and in vivo. However, the underlying mechanism(s) of LCAT1 as an oncogenic regulator remains elusive. Here, we showed that LCAT1 physically interacts with and stabilizes IGF2BP2, an m^6^A reader protein, by preventing its degradation via autolysosomes. IGF2BP2 is overexpressed in lung cancer tissues, which is associated with poor survival of non-small cell lung cancer patients, suggesting its oncogenic role. Biologically, IGF2BP2 depletion inhibits growth and survival as well as the migration of lung cancer cells. Mechanistically, the LCAT1/IGF2BP2 complex increased the levels of CDC6, a key cell cycle regulator, by stabilizing its mRNA in an m^6^A-dependent manner. Like IGF2BP2, CDC6 is also overexpressed in lung cancer tissues with poor patient survival, and CDC6 knockdown has oncogenic inhibitory activity. Taken together, the LCAT1-IGF2BP2-CDC6 axis appears to play a vital role in promoting the growth and migration of lung cancer cells, and is a potential therapeutic target for lung cancer. Importantly, our finding also highlights a previously unknown critical role of LCAT1 in m^6^A-dependent gene regulation by preventing autolytic degradation of IGF2BP2.

## Introduction

Lung cancer is the second most commonly diagnosed malignant cancer and the primary cause of cancer-related mortalities worldwide, with 2,206,771 new cases of lung cancer and 1,796,144 deaths resulting from lung cancer in 2020 [1]. Despite advances in surgery and treatment strategies over recent decades, including target therapy and immunotherapy, the overall survival rate of patients with lung cancer remains poor [2]. Further and fuller understanding of the molecular mechanisms that promote lung cancer initiation and progression are still required to formulate yet more effective treatment strategies.

Long non-coding RNAs (lncRNAs) are a class of non-coding RNAs whose transcripts are of more than 200 nucleotides in length and make up most of the human transcriptome [3]. LncRNAs participate in almost all spheres of human biology but most notable here is their strong role in tumor development [4–6]. It has been reported that several lncRNAs, including MALAT1, TUG, NEAT1, and LCAT3 are dysregulated in lung cancers, acting to modulate lung tumor development and progression [7–9]. Our recent study provided an example of this, showing that LCAT3 recruits FUBP1 to the FUSE region of the MYC promoter, thereby activating MYC transcription to promote lung cancer cell growth and metastasis [10]. However, the specific functions and molecular mechanisms of most other lncRNAs in lung cancer remain elusive.

Insulin-like growth factor 2 mRNA-binding protein 2 (IGF2BP2) plays a vital role in the post-transcriptional regulation of RNAs [11]. N6-methyladenosine (m6A) modification is the most prevalent internal RNA modification in eukaryotic cells [12, 13]. A recent study has shown that IGF2BP2 functions as a reader protein for m6A modification [14]. The fate of m6A-modified RNAs rely on the functions of different proteins that recognize them (such as IGF2BP1–3, YTHDF1–3, and YTHDC1–3) [14–16]. These m6A reader proteins may affect the splicing [17], stability [12] and/or translation of targeted RNAs [18]. These findings expand the understanding of the function of IGF2BP2 in contributing to tumorigenesis and progression. However, the specific role of IGF2BP2 in lung cancer remains to be investigated.

We recently identified a novel lncRNA, LCAT1, which is overexpressed in lung cancer and correlated with poor prognosis in lung cancer patients. Biologically, LCAT1 promotes the growth and metastasis of lung cancer cells both in vitro and in vivo [19], but the underlying mechanism(s) by which LCAT1 acts as an oncogenic regulator remains to be thoroughly defined. In the present study, by employing RNA pull-down, mass spectrometry (MS) and RNA immunoprecipitation (RIP) assays, we found that LCAT1 physically interacts with IGF2BP2 and stabilizes IGF2BP2 by protecting it from autolytic degradation. Biologically, IGF2BP2 knockdown suppressed growth, colony formation and migration of lung cancer cells. Mechanistically, the LCAT1/IGF2BP2 complex stabilized CDC6 mRNA via the m^6^A modification to increase the translation of CDC6, which then promoted the growth and survival of lung cancer cells. Taken together, our study revealed that the LCAT1-IGF2BP2-CDC6 axis has oncogenic activity and may serve as a potential therapeutic target as well as a prognostic biomarker for lung cancer.

## Materials and methods

### Cell lines and cell culture

The human lung cancer cell lines A549, Calu1, and HOP62 were obtained from the American Type Culture Collection (Manassas, VA, USA). They were cultured in RPMI-1640 medium (Gibco, Carlsbad, CA, USA) supplemented with 10% fetal bovine serum (FBS, Thermo Fisher Scientific, Rockford, IL, USA) in a humidified atmosphere with 5% CO_2_ at 37 °C. The HEK-293T cell line was grown in Dulbecco’s modified essential medium (DMEM, Gibco) supplemented with 10% FBS in a humidified atmosphere with 5% CO_2_ at 37 °C.

### Reagents and antibodies

The reagents and antibodies used in this study are listed in Supplementary Table 1.

### siRNA and transfection

The small interference RNAs (siRNAs) targeting IGF2BP2 and CDC6 (Supplementary Table 2) were designed using the website: http://rnadesigner.thermofisher.com/ and synthesized by RiboBio (Guangzhou, China). All siRNAs were transfected into lung cancer cells at a final concentration of 50 nM using GenMute^TM^ reagent according to the manufacturer’s instructions.

### RNA isolation and quantitative RT-PCR

Total RNA from frozen human lung cancer tissues, adjacent tissues, and cultured cells were extracted using Trizol reagent (Invitrogen, Carlsbad, CA, USA). The cDNA was then synthesized using HiScript® II Reverse Transcriptase (Vazyme, Nanjing, China) according to the manufacturer’s instructions. qRT-PCR was performed using an SYBR Green PCR Mix Kit (Vazyme).

### Cell proliferation and colony assays

Cell proliferation and colony assays were conducted as previously reported [19]. For the proliferation assay, cells were seeded into 96-well plates with a density of 1000 cells/well. CCK-8 reagents (MCE, Monmouth Junction, NJ, USA) were then added into cells at the indicated time and incubation about 2 h before the OD measurement. Cell proliferation was also assessed using a Cell-Light EdU DNA cell proliferation kit (RiboBio, Guangzhou, China), following the manufacturer’s instructions. For the colony assay, 1500 control cells and the treated cells were seeded into 12-well plates and cultured for one week. Colonies were fixed with methanol and stained with 0.1% crystal violet.

### Migration assay

Cell migration assays were conducted by using transwell chambers (Corning Costar, Tewksbury, MA, USA). Briefly, 3 × 10^4^ cells were seeded into the upper chamber of each insert with 300 μl serum-free medium and 500 μl medium with 10% FBS added into the lower chambers. After incubating at 37 °C for 12 h, the cells were fixed and stained with 0.1% crystal violet. Cell numbers were counted in three random areas.

### Western blot analysis

Cells were lysed in RIPA buffer and proteins were quantified using a Pierce^TM^ BCA Protein Assay Kit (Thermo Scientific). Equal amounts of proteins were loaded and separated in a 10% polyacrylamide SDS gel, and then transferred to a PVDF membrane (Millipore, Burlington, MA, USA). After incubation with the intended primary antibody and secondary antibody, proteins were detected using Pierce ECL Western Blotting Substrate reagent (Thermo Scientific).

### RNA pull-down

Biotin-labeled targeted LCAT1 sense and antisense probes were synthesized by Life Technologies (Carlsbad, CA, USA). The RNA pull-down assay was conducted by using a Pierce™ Magnetic RNA-Protein Pull-Down Kit (Thermo Scientific) according to the user guide. In brief, 50 pmol biotin-labeled probes were incubated with streptavidin beads for 1 h at room temperature. Cell lysate from Calu1 cells were then added to the beads and incubated overnight at 4 °C. Subsequently, the proteins were eluted from the beads and separated by 10% SDS-PAGE followed by silver staining.

### RNA immunoprecipitation (RIP) assay

RIP was performed using a Magna RIP Kit (Millipore) following the manufacturer’s instructions. Briefly, a total of 5 × 10^6^ cells were lysed in 115 μl RIP lysis buffer for 10 min. Five micrograms of the intended antibody was used for each immunoprecipitation assay and was conjugated to protein A/G magnetic beads by incubation for 1 h at room temperature, followed by washing three times with RIP wash buffer and incubation with RIP lysates overnight at 4 °C. RNA was then extracted by phenol, chloroform and isoamyl alcohol according to the user guide, and was quantitated by real-time PCR.

### RNA-seq and data analysis

The RNA sequencing library was prepared as described previously [20]. Briefly, total RNA was extracted from Calu1 transfected with IGF2BP2 siRNA (si-IGF2BP2 1# or si-IGF2BP2 2#) or Control siRNA (*n* = 3). mRNA libraries were constructed using the TruSeq Sample Preparation Kit (Illumina). The mRNA libraries were multiplexed and sequenced using an Illumina HiSeq X10 sequencer. Transcriptome data were analyzed as previously described (Supplementary Table 3) [21].

### Enrichment analysis of IGF2BP2 targets

IGF2BP2 functions as a reader protein for m6A modification [14]. Therefore, we downloaded the list of IGF2BP2 targets that were identified by PAR-CLIP and RIP-seq [14, 22]. Of these IGF2BP2 targets, 2573 transcripts were expressed in lung cancer cell lines. We then assessed if these IGF2BP2 targets are enriched in the list of genes that are altered by the knockdown of either LCAT1 or IGF2BP2 in lung cancer cells. We plotted the cumulative distribution of fold changes (knockdown versus control) for IGF2BP2 targets and non-IGF2BP2 targets using the “ggecdf” function of the R package “ggpubr”. The difference in the cumulative distribution of log2(fold change) between IGF2BP2 targets and non-IGF2BP2 targets was examined by Kolmogorov–Smirnov test.

### m^6^A dot blot

The m6A dot assay was performed as previously described [23]. Briefly, RNA was diluted to a certain concentration and denatured by incubation at 95 °C for 5 min. Denatured RNA was then loaded onto a nylon membrane (GE Healthcare, Chicago, IL, USA) followed by UV crosslinking. The membrane was then blocked in 5% non-fat milk for 1 h at room temperature. Subsequently, the membrane was incubated with an anti-m6A antibody (1:1000; Synaptic Systems) overnight at 4 °C. After incubation with anti-rabbit IgG secondary antibody (Cell Signal Technology), the membrane was detected using the ECL system (Bio-Rad, Hercules, CA, USA). Finally, the membrane was stained with methylene as a loading control.

### Gene-specific m^6^A qPCR

mRNA was purified from total RNA by using a polyA Spin^TM^ mRNA Isolation Kit (NEB, Ipswich, MA USA). m6A modifications on target genes were detected using a Magana MeRIP m6A Kit (Millipore) according to the manufacturer’s instructions. In brief, 18 μg of purified mRNA was sheared into ~100 nt oligonucleotides in a fragmentation buffer and then incubated with anti-m6A antibody (Synaptic Systems)-conjugated or normal mouse IgG-conjugated beads at 4 °C overnight. Eluted RNA was then prepared for MeRIP-qPCR analysis.

### RNA stability assay

For the RNA stability assay, lung cancer cells transfected with IGF2BP2 and LCAT1 siRNA were treated with 5 μg/mL actinomycin D (Sigma-Aldrich, St. Louis, MO, USA) to suppress transcriptional activity. Treated cells were collected at different time points, followed by RNA isolation and qPCR.

### Animal experiments

For the in vivo tumorigenicity assay, female BALB/c nude mice (ages 4–5 weeks) were randomly divided into two groups (*n* = 5 per group). A549 cells (2 × 10^6^) that had been stably transfected with sh-LCAT1 or scramble were implanted subcutaneously into the nude mice. Tumor growth was measured once a week and tumor volumes were calculated with the following formula: Volume (cm^3^) = (length × width^2^)/2. After 4 weeks, the mice were euthanized and tumors were collected and weighed. All experiments were performed in accordance with the Guide for the Care and Use of Laboratory Animals (NIH publication 80-23, revised 1996), with the approval of Zhejiang University, Hangzhou, China.

### Survival analysis

RNA-seq data for IGF2BP2 and CDC6 in lung adenocarcinoma samples from The Cancer Genome Atlas (TCGA) were downloaded from the ICGC Data Portal (https://dcc.icgc.org). Clinical information such as overall survival (OS) and disease-free survival (DFS) time for these TCGA patients was also retrieved from the ICGC Data Portal. To further evaluate the clinical significance of IGF2BP2 and CDC6, two additional public lung cancer datasets (GSE30219 and GSE81089) with complete clinical information and commonly used in previous studies were downloaded from Gene expression Omnibus (GEO) databases [24, 25]. Patients were divided into two groups according to the median expression of IGF2BP2 or CDC6. Survival distributions in two different groups were visualized using Kaplan–Meier curves. Differences in OS and DFS between the two groups were assessed by a log-rank test.

### Statistical analysis

All statistical analyses were performed using the R Statistical Package. Results were obtained from at least three independent experiments and data were presented as the mean ± SD. For comparisons of two groups, two-tailed paired Student’s *t*-tests were conducted. Comparison of multiple groups was made using a one- or two-way ANOVA.

## Results

### LCAT1 physically interacts with and stabilizes IGF2BP2, an m^6^A reader protein

**Fig. 1:**
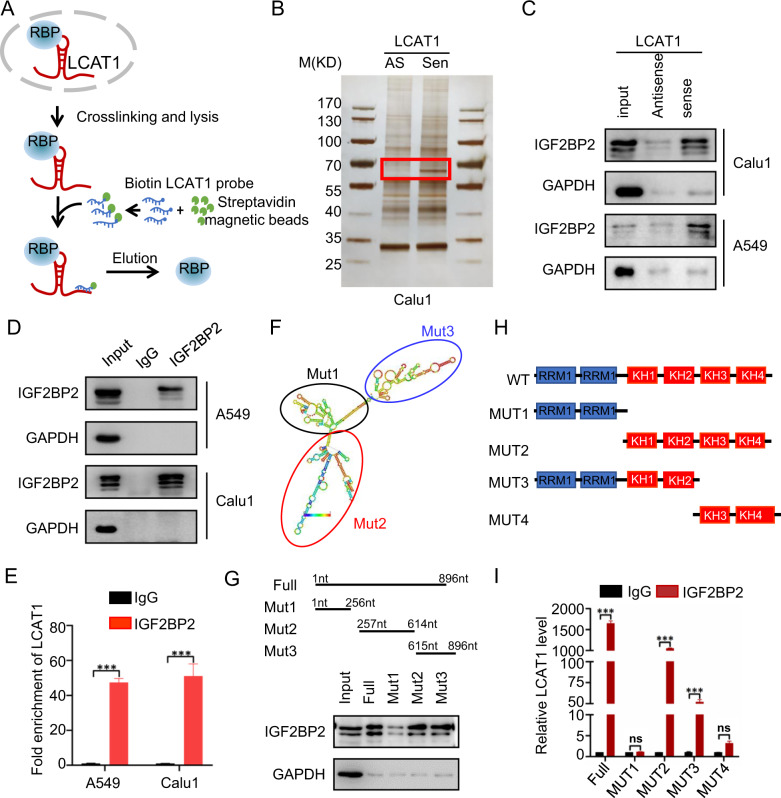
LCAT1 physically interacts with the m^6^A reader protein IGF2BP2 in lung cancer cells. **A** Schematic diagram of RNA pull-down assay. **B** Proteins were pull-downed by LCAT1 antisense/sense probes followed by silver staining. **C** Immunoblotting to confirm the specific interaction between LCAT1 and IGF2BP2. GAPDH was used as a negative control. **D**, **E** RIP assays were performed using the anti-IGF2BP2 antibody to reversely verify the LCAT1-IGF2BP2 interaction. qPCR was used to determine LCAT1 enrichment. **F** The secondary structure of LCAT1 predicted by *RNAfold* webserver. **G** Western blot was used to detect IGF2BP2 protein pulled down by different mutants of LCAT1. **H** Structural diagram of different IGF2BP2 mutant proteins. **I** RIP assays were conducted using anti-FLAG antibodies to detect the enrichment of LCAT1 in different IGF2BP2 mutants. **H** Western blot was used to detect the IGF2BP2 protein level in LCAT1 knockdown and control cells. The data were presented as the mean ± SD; *n* = 3; Student’s two-sided *t*-test were used, *P < 0.05, **P < 0.01, ***P < 0.001.

To further explore the mechanism by which LCAT1 acts as an oncogenic regulator in lung cancer cells, we conducted RNA pull-down, followed by silver staining to identify potential LCAT1 binding proteins (Fig. 1A). This effort led to the identification of a distinct protein band of ~70 kDa, detected only in LCAT1 sense probe (Fig. 1B). This band was then cut from the gel for MS analysis. Among the highly enriched proteins, the well-known RNA and DNA binding protein IGF2BP2 drew our attention for its prominent role as an m^6^A reader in tumorigenesis (Supplementary Table 4). Western blotting assays confirmed that IGF2BP2 was significantly enriched in the sense-LCAT1 but not the antisense-LCAT1 pull-down fraction in both lung cancer cell lines (Fig. 1C). This interaction was further verified by RNA immunoprecipitation (RIP) assay with anti-IGF2BP2 or anti-IgG antibodies. The amount of LCAT1 RNA in the coprecipitation was measured by qPCR and showed that LCAT1 was significantly enriched in the IGF2BP2 RIP group compared with the IgG group (Fig. 1D, E).

In addition, to identify the specific region of LCAT1 that interacts with IGF2BP2, we constructed three LCAT1 deletion mutants based on the secondary structure of LCAT1 predicted by *RNAfold* Webserver (Fig. 1F). IGF2BP2 mainly binds to the region between 257–896 nucleotides of LCAT1 (Fig. 1G). Likewise, a series of IGF2BP2 truncation mutants were constructed based on the domain structure of IGF2BP2 (Fig. 1H). FLAG-tagged-based pull-down showed that LCAT1 mainly binds to the KH domain of IGF2BP2 (Fig. 1I). Collectively, these results indicated that LCAT1 physically interacts with the m^6^A reader protein IGF2BP2.

### LCAT1 regulates IGF2BP2 protein stability

**Fig. 2:**
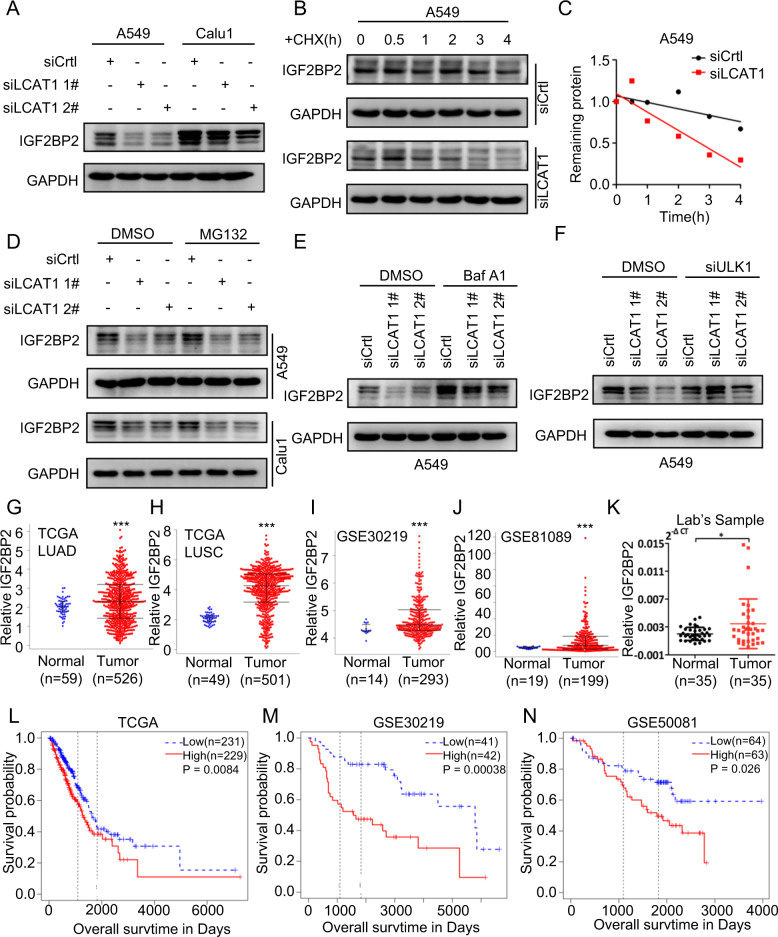
LCAT1 regulates IGF2BP2 protein stability, and IGF2BP2 is upregulated and associated with poor survival in lung cancer patients. **A** Western blot was used to detect the IGF2BP2 protein level in LCAT1 knockdown and control cells. **B**, **C** Stability of IGF2BP2 protein in LCAT1 knockdown and control cells, with or without CHX treatment. **D** IGF2BP2 protein levels in LCAT1 knockdown and control cells, with or without treatment of the protease inhibitor MG-132. **E** IGF2BP2 protein levels in LCAT1 knockdown and control cells treated with different concentrations of the autophagy inhibitor Bafilomycin A1. **F** IGF2BP2 protein levels in ULK1 knockdown and control cells. **G**–**J** The relative expression level of IGF2BP2 in TCGA-LUAD, TCGA-LUSC, GSE30219, and GSE81089, respectively. **K** qPCR was performed to detect IGF2BP2 expression in 35 fresh lung tumor tissues and matched adjacent non-tumor tissues. **L**–**N** Kaplan–Meier curves of overall survival of lung cancer patients based on IGF2BP2 mRNA expression. Data were presented as mean ± SD. *P < 0.05; **P < 0.01; ***P < 0.001.

Then, we measured the IGF2BP2 levels after LCAT1 knockdown. Interestingly, we found that LCAT1 knockdown significantly reduced IGF2BP2 protein levels, whereas IGF2BP2 knockdown had little, if any, effect on LCAT1 expression (Fig. 2A and Supplementary Fig. 1A, B). We then determined whether LCAT1 binding would affect IGF2BP2 stability by cycloheximide (CHX) treatment to block new protein synthesis, and found that LCAT1 knockdown significantly shortened IGF2BP2 protein half-life (Fig. 2B, C). However, treatment with the proteasome inhibitor MG-132 could not restore reduced IGF2BP2 levels in LCAT1 knockdown cells (Fig. 2D), suggesting IGF2BP2 reduction was not via enhanced degradation by the ubiquitin-proteasome system (UPS).

It is well-known that cellular proteins can also be degraded through the lysosome pathway [26, 27]. Notably, we found that endogenous IGF2BP2 levels in lung cancer cells were significantly increased by various autophagy inhibitors, including Bafilomycin A1, 3-MA and NH_4_Cl (Supplementary Fig. 1C), but remarkably reduced by autophagy activators Rapamycin, AZD8055 and EBSS (Supplementary Fig. 1D), strongly suggesting that basal levels of IGF2BP2 are dynamically regulated by a lysosomal system in lung cancer cells. More importantly, IGF2BP2 reduction upon LCAT1 knockdown was completely rescued by Bafilomycin A1 (Fig. 2E) as well as by siRNAs targeting ULK1, a key regulator of autophagy [28, 29] (Fig. 2F and Supplementary Fig. 1E, F). Taken together, these results demonstrated that LCAT1 binds to and stabilizes IGF2BP2 via blockage of lysosome degradation.

Previous studies have shown that IGF2BP2 is frequently dysregulated in multiple human cancers [30–32]. IGF2BP2, for example, modulated lncRNA DANCR expression in pancreatic cancer to promote cell proliferation by acting as an m^6^A reader [33]. However, whether and how IGF2BP2 affects lung cancer cells remains to be determined. We first evaluated the clinical significance of IGF2BP2 in human cancers and analyzed RNA-seq data from TCGA projects. It was found that IGF2BP2 was widely upregulated in a variety of cancers, including head and neck, cervical and uterine, esophageal (Supplementary Fig. 2A), and lung cancers (Fig. 2G, H). The upregulation of IGF2BP2 in lung cancer was consistently verified using different datasets (Fig. 2I, J). Then, we evaluated the mRNA levels of IGF2BP2 in 35 fresh lung cancer tissues and their adjacent normal tissues by qPCR. The qPCR result confirmed that the expression of IGF2BP2 was significantly increased in lung cancer tissues compared with adjacent normal tissues (Fig. 2K and Supplementary Fig. 2B). More importantly, lung cancer patients with higher expression of IGF2BP2 were associated with shorter overall survival and disease-free survival time in TCGA dataset (Fig. 2L and Supplementary Fig. 2C). It was also observed that patients with higher expression of IGF2BP2 tended to have a worse prognosis in the Gene expression Omnibus (GEO) datasets (Fig. 2M, N). Overall, these results demonstrate that IGF2BP2 is upregulated and associated with poor outcomes in lung cancer.

### IGF2BP2 promotes the growth, survival, and migration of lung cancer cells

**Fig. 3:**
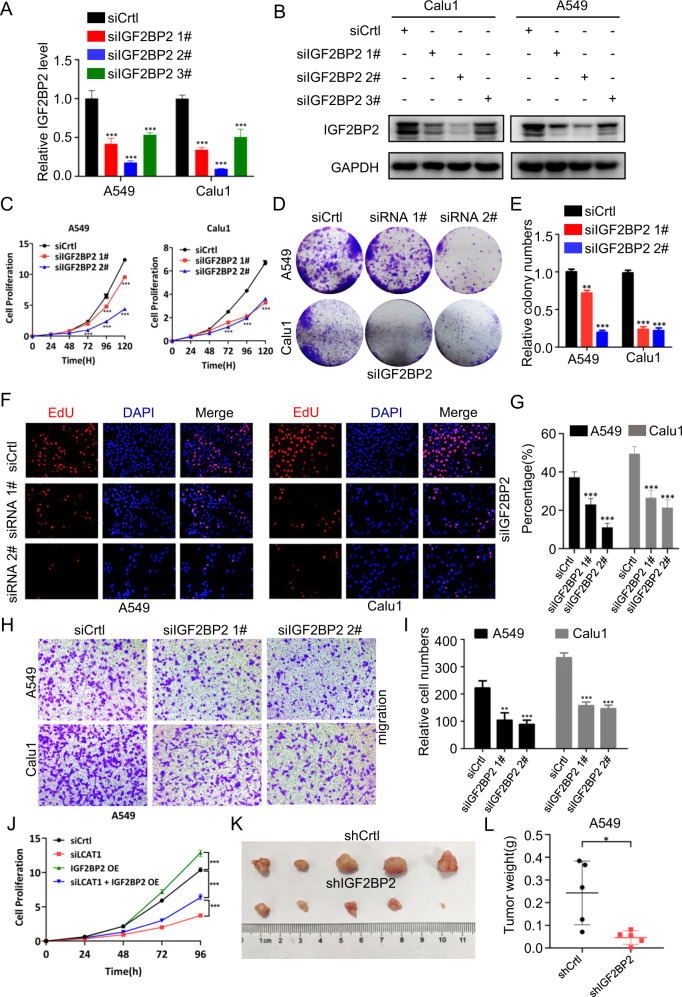
IGF2BP2 promotes lung cancer cell proliferation and progression. **A**, **B** qPCR and western blot analyses confirmed that IGF2BP2 was successfully knocked down by siRNA in A549 and Calu1 cells. **C**–**E** CCK-8 and colony formation assays in the A549 and Calu1 cells with IGF2BP2 knockdown. **F**, **G** EdU assays were conducted to evaluate the DNA replication in IGF2BP2 silenced cells. **H**, **I** Transwell assays were performed to evaluate the impacts of IGF2BP2 on lung cancer cell migration. **J** CCK-8 assays showed that overexpression of IGF2BP2 could rescue the cell proliferation resulting from the inhibition of LCAT1. **K**, **L** Xenograft subcutaneous tumors formed by IGF2BP2-knockdown A549 cells are smaller than those formed by control cells. Student’s two-sided *t*-tests were used. *P < 0.05, **P < 0.01, ***P < 0.001.

We next determined the biological function of IGF2BP2 and found that IGF2BP2 knockdown in lung cancer cells (Fig. 3A, B) significantly suppressed the growth (Fig. 3C) and colony formation (Fig. 3D, E), whereas ectopic IGF2BP2 expression (Supplementary Fig. 3A, B) moderately promoted cell growth and survival (Supplementary Fig. 3C, D). We then investigated the cell cycle transition by flow cytometry and found that IGF2BP2 knockdown resulted in the accumulation of cell populations in the G0/G1 phase (Supplementary Fig. 3E, F). Consistently, the expression levels of some key regulators of the cell cycle, such as CyclinB1, CyclinD1, and p27, were altered (Supplementary Fig. 3G). In addition, the EdU-based proliferation assay also showed that IGF2BP2 knockdown inhibited DNA replication, as a readout of reduced cell proliferation (Fig. 3F, G). IGF2BP2 knockdown also significantly inhibited the migration capacity of lung cancer cells (Fig. 3H, I). We then directly examined the biological cross-talk between LCAT1 and IGF2BP2, and found that growth suppression, triggered by LCAT1 knockdown, as we previously shown [19], could be largely reversed by IGF2BP2 overexpression (Fig. 3J), suggesting that the biological effect of LCAT1 on cancer cell growth is mediated, at least in part, through IGF2BP2. Finally, we evaluated the effect of IGF2BP2 knockdown on the in vivo tumorigenicity using a xenograft nude mouse model. A549 lung cancer cells with stable IGF2BP2 knockdown (Supplementary Fig. 3H, I) showed significantly suppressed growth, reflected by reduced tumor size and weight (Fig. 3K, L). Taken together, IGF2BP2 is required for the growth and survival of lung cancer cells, supporting the notion that IGF2BP2 could be an attractive therapeutic target for lung cancer treatment.

### CDC6 is a downstream target of the LCAT1/IGF2BP2 complex

To elucidate the underlying mechanism by which IGF2BP2 affects the growth and survival of lung cancer cells, we employed the RNA-seq technology to reveal potential pathways altered upon IGF2BP2 knockdown in lung cancer cells, given the fact that IGF2BP2 is an m^6^A reader, essential for the stability, location and translation of target RNAs [14], with high-likelihood on transcription regulation. Indeed, unbiased transcriptome analysis showed that a large number of genes were significantly either upregulated or downregulated in response to IGF2BP2 knockdown (Supplementary Fig. 4A). These genes, potentially regulated by IGF2BP2, were enriched in many important cancer-related pathways (Supplementary Fig. 4B, C).

**Fig. 4:**
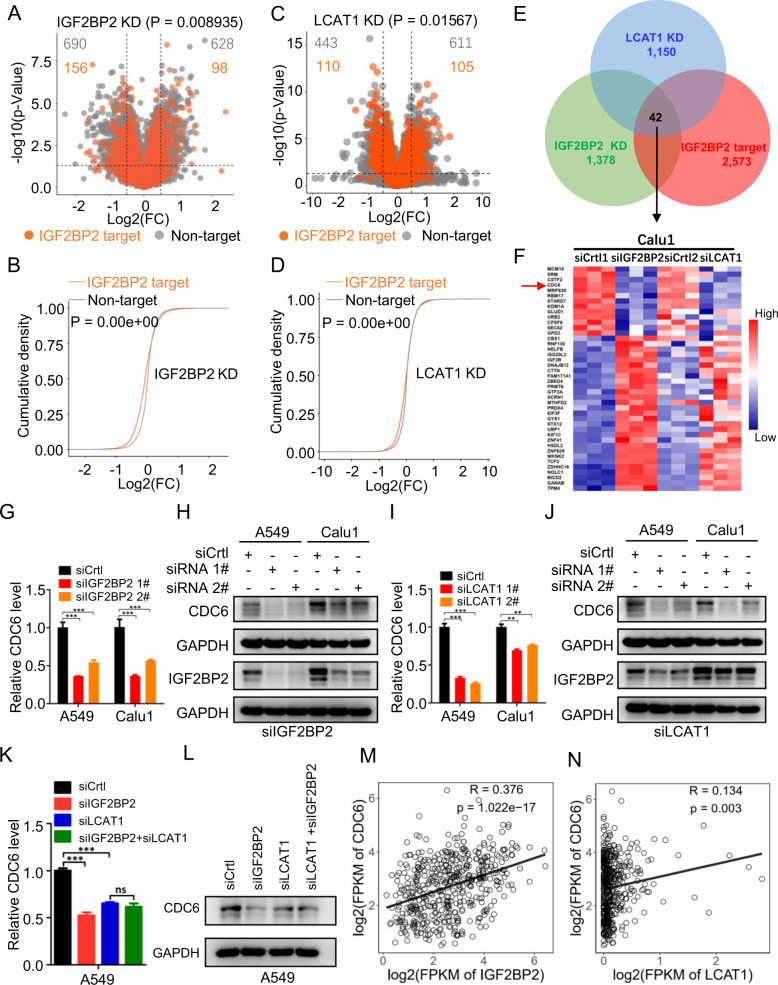
CDC6 is a downstream target of the LCAT1/IGF2BP2 complex. **A**, **C** Volcano plots showing enrichment of IGF2BP2 target genes that were altered in IGF2BP2 knockdown (**A**) or LCAT1 knockdown (**C**) Calu1 cells. The numbers of significantly downregulated (log2 FC < −1, *P* < 0.05, two-sided Student’s *t*-test) or upregulated (log2 FC > 1, *P* < 0.05, two-sided Student’s *t*-test) genes in the IGF2BP2 target group and non-target group are displayed. Vertical dashed lines indicate the cut-off of log2 FC of 1 or –1, while the horizontal dashed line indicates the cut-off of a *P* value of 0.05. FC, fold change. **B**, **D** Cumulative density of mRNA log2 FC in IGF2BP2 target and non-target genes upon IGF2BP2 (**B**) or LCAT1 (**D**) knockdown. **E** Venn diagram of the cross-comparison of three datasets: RNA-seq of IGF2BP2 knockdown; RNA-seq of LCAT1 knockdown; and IGF2BP2 targets. **F** Heatmap of target genes. **G**, **H** qPCR and western blot were performed to detect CDC6 expression in IGF2BP2 knockdown and control cells. **I**, **J** qPCR and western blot were performed to detect CDC6 expression in LCAT1 knockdown and control cells. **K**, **L** qPCR and western blot were conducted to determine the impacts of LCAT1 and IGF2BP2 silencing on CDC6 expression. **M** Scatter plot of IGF2BP2 and CDC6 mRNA expression in lung cancers from the TCGA. **N** Scatter plot of LCAT1 and CDC6 mRNA expression in lung cancers from the TCGA. Student’s two-sided *t*-tests were used. *P < 0.05, **P < 0.01, ***P < 0.001.

We next downloaded the list of IGF2BP2 targets that were identified by PAR-CLIP and RIP-seq [14, 22]. Of these IGF2BP2 targets, 2573 transcripts were detected in lung cancer cells. We compared the expression changes (represented by log2(fold change)) of IGF2BP2 targets (i.e., 2573 transcripts) and non-IGF2BP2 targets in lung cancer cells with IGF2BP2 knockdown. Interestingly, IGF2BP2 targets had a significantly higher proportion of negative log2(fold change) values than non-IGF2BP2 targets, implying that knockdown of IGF2BP2 globally and preferentially inhibited the expression of IGF2BP2 target genes in lung cancer cells upon IGF2BP2 knockdown (Fig. 4A, B). Given the physical association between LCAT1 and IGF2BP2, and the positive regulation of IGF2BP2 by LCAT1, we found that LCAT1 depletion also preferentially inhibited the expression of IGF2BP2 targets (Fig. 4C, D). We then performed an integrative analysis of RNA-seq of cells with knockdown of either LCAT1 or IGF2BP2. This yielded a total of 42 IGF2BP2 targets that were simultaneously altered in cells with either LCAT1 or IGF2BP2 knockdown (Fig. 4E). Thirteen of 42 targets were downregulated both in LCAT1 and IGF2BP2 knockdown cells (Fig. 4F). Among them, the top five downregulated genes were selected for further evaluation by qPCR assay. The qPCR results indicated that cell division cycle 6 (CDC6) was the most significantly downregulated gene when LCAT1 or IGF2BP2 was silenced, which was chosen for further investigation (Supplementary Fig. 4D–G).

We then measured the levels of CDC6 in lung cancer cells with either IGF2BP2 or LCAT1 knockdown. Expectedly, both the mRNA and protein levels of CDC6 were dramatically decreased upon knockdown of IGF2BP2 (Fig. 4G, H) or LCAT1 (Fig. 4I, J). Conversely, the levels of CDC6 were increased upon ectopic expression of either IGF2BP2 or LCAT1 (Supplementary Fig. 4H, I). Combinational knockdown of both IGF2BP2 and LCAT1 did not further decrease the levels of either CDC6 mRNA or CDC6 protein (Fig. 4K, L), indicating that CDC6 is subjected to positive regulation by the lineal LCAT1-IGF2BP2 axis.

Finally, we analyzed RNA-seq data from TCGA to further investigate the correlation between CDC6 and IGF2BP2 or LCAT1. It was found that the CDC6 expression was positively correlated with IGF2BP2 or LCAT1, but to a less extent (Fig. 4M, N). Taken together, CDC6 appears to be a direct target downstream of the LCAT1/IGF2BP2 complex for positive regulation with oncogenic potential in lung cancer cells.

### CDC6 is upregulated and promotes the growth of lung cancer cells

CDC6 is a known key player in modulating DNA replication and maintaining cell cycle checkpoint control [34, 35], and is also actively involved in tumorigenesis [36, 37]. For instance, CDC6 was shown to exert its oncogenic activity by repressing the transcription of E-cadherin and activating adjacent replication origins [38]. CDC6 is highly expressed in various human cancers, including lung cancer (Supplementary Fig. 5A–E). The upregulation of CDC6 was further verified in our collection of paired lung cancer tissues (*n* = 35), using qPCR assay (Supplementary Fig. 5F). Importantly, lung cancer patients with higher CDC6 expression have the worse clinical outcome (Supplementary Fig. 5G–I).

**Fig. 5:**
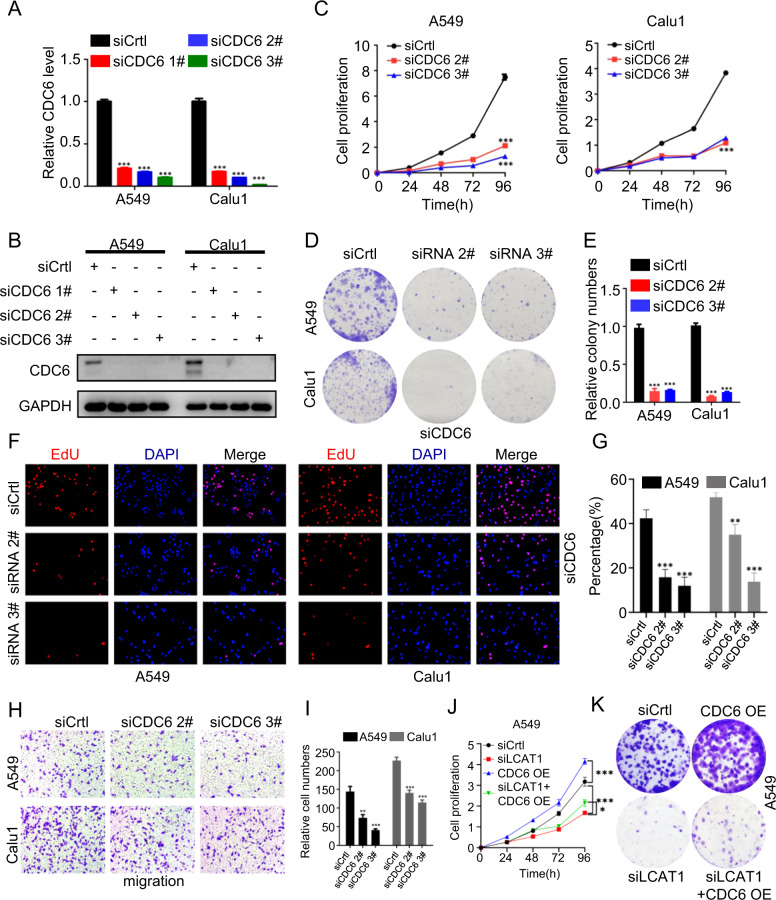
CDC6 knockdown inhibits lung cancer cell proliferation, colony formation, and migration. **A**, **B** qPCR and western blot were taken to measure the CDC6 expression in A549 and Calu1 cells transfected with CDC6 siRNAs and negative control. **C**–**G** CCK-8 (**C**), colony formation (**D**, **E**), and EdU staining (**F**, **G**) assays were performed to evaluate the impacts of CDC6 depletion on lung cancer cell viability. **H**, **I** Transwell assays were conducted to determine the migration ability of CDC6 silenced and control cells. **J**, **K** Growth curve and colony formation assays were performed in A549 cells co-transfected with siLCAT1 and CDC6 overexpress vector. *P < 0.05, **P < 0.01, ***P < 0.001.

As mentioned above, CDC6 is a downstream target of the LCAT1-IGF2BP2 axis and is upregulated in lung cancer tissues. Next, we determined the biological consequence of CDC6 knockdown in lung cancer cells. Indeed, upon CDC6 knockdown in A549 and Calu1 cells (Fig. 5A, B), cell proliferation (Fig. 5C) and colony formation ability were significantly decreased based on CCK-8 and colony formation assays (Fig. 5D, E). As expected, when CDC6 was knocked down, the proportion of S-phase cells was apparently reduced (Supplementary Fig. 6A, B) and several cell cycle-related proteins such as CyclinB1, CyclinD1, and CDK2 were correspondingly significantly downregulated (Supplementary Fig. 6C). Consistently, EdU assays showed that CDC6 depletion inhibited DNA replication in lung cancer cells (Fig. 5F, G). In addition, the silencing of CDC6 resulted in a marked reduction in cell migration ability (Fig. 5H, I). Then, the rescue experiment was performed to validate the specificity of the LCAT1-IGF2BP2-CDC6 axis. Forced expression of CDC6 could overcome the cellular effect caused by LCAT1 knockdown (Fig. 5J, K and Supplementary Fig. 6D, E). These results suggest that the effect of the LCAT1-IGF2BP2 axis may be mediated, at least in part, by CDC6.

### LCAT1/IGF2BP2 complex enhances CDC6 mRNA stability in an m^6^A-dependent manner

**Fig. 6:**
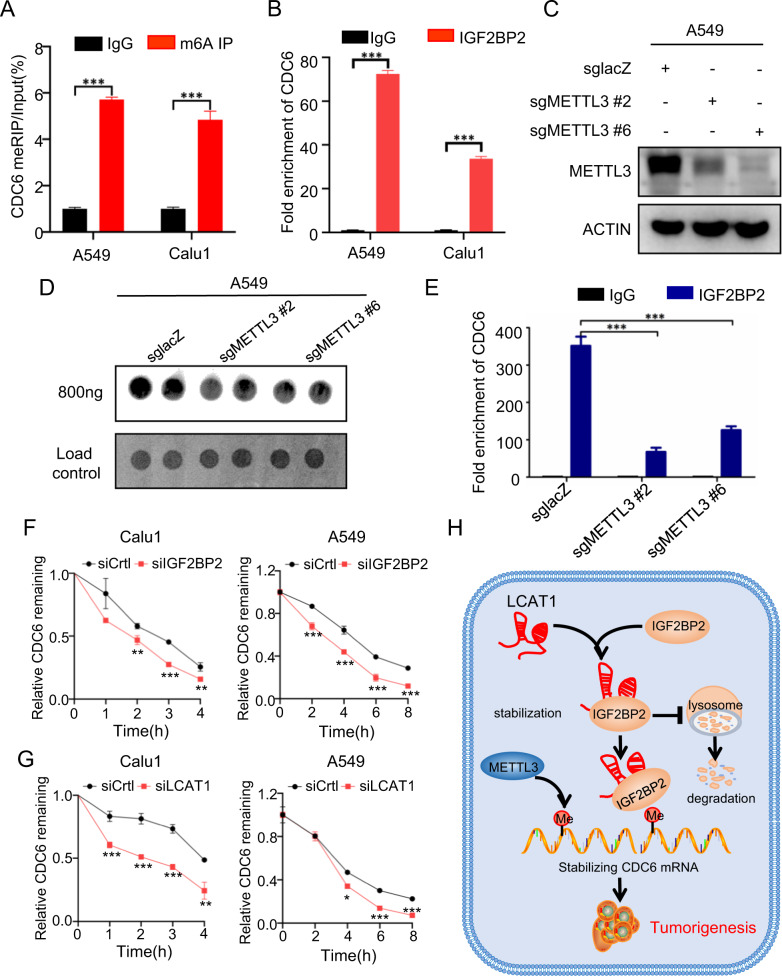
LCAT1/IGF2BP2 enhances CDC6 mRNA stability via an m^6^A-dependent manner. **A** MeRIP-qPCR was performed to determine the enrichment of m^6^A-modified CDC6 RNA in anti-m^6^A and IgG groups. **B** RIP-qPCR was conducted to evaluate the enrichment of CDC6 in anti-IGF2BP2 antibody and IgG antibody groups. **C** Western blot of METTL3 knockout in A549 cells. **D** Dot blot assay indicated that knockout of METTL3 decreased global levels of m^6^A modification. **E** RIP-qPCR was performed in METTL3 knockout A549 cells using an anti-IGF2BP2 antibody or IgG antibody. **F**, **G** Depletion of IGF2BP2 (**F**) or LCAT1 (**G**) reduced CDC6 RNA stability in lung cancer cells. Cells were first transfected with IGF2BP2 siRNA or LCAT1 siRNA, then treated with 2 μg/ml actinomycin D at different time points. **H** Schematic diagram of the LCAT1-IGF2BP2-CDC6 axis in mediating lung cancer pathogenesis. *P < 0.05, **P < 0.01, ***P < 0.001.

We next elucidated the underlying mechanism by which the LCAT-IGF2BP2 axis stabilizes the CDC6 level. Given that IGF2BP2 is an m^6^A reader, known to regulate the stability of its target RNAs and the rate of mRNA translation [14], we, therefore, determined whether IGF2BP2 regulation of CDC6 is in a manner dependent on the m^6^A modification. The MeRIP-qPCR assays in A549 and Calu1 cells confirmed that the CDC6 mRNA was much enriched using the m^6^A antibody group, compared to the IgG control (Fig. 6A), indicating that CDC6 mRNA is subjected to m^6^A modifications. Next, we conducted RIP assays using the anti-IGF2BP2 antibody and found that the CDC6 mRNA was notably enriched (Fig. 6B). m6A modifications are deposited by m6A methyltransferases complex (MTC; i.e., m6A writer), which is composed of METTL3, METTL14, WTAP, and possibly VIRMA and RBM15. Among them, METTL3 is the core catalytic subunit of MTC and is essential for m6A modification [13, 39]. Therefore, we chose METTL3, the core m6A writer, to investigate whether the interaction between IGF2BP2 protein and CDC6 mRNA is dependent on m^6^A modifications. We employed the CRISPR/Cas9 system to knockout METTL3 in A549 cells and generated two stable knockdown clones (2# and 6#) (Fig. 6C). Subsequent dot blot assays confirmed that METTL3 knockdown cells indeed had decreased total m^6^A levels (Fig. 6D). Remarkably, the enrichment of CDC6 mRNA in the IGF2BP2 immunoprecipitation was abolished upon METTL3 knockdown (Fig. 6E), strongly suggesting that the IGF2BP2-CDC6 mRNA interaction is largely dependent of the m^6^A modification of CDC6 mRNA. Finally, we determined whether the CDC6 mRNA stability is indeed affected by the knockdown of either LCAT1 or IGF2BP2 in two lung cancer cell lines. The knockdown cells were treated with actinomycin D (to block new mRNA synthesis) for intended hours, and the CDC6 mRNA level was then measured by qPCR at each time point. The results showed that depletion of either IGF2BP2 or LCAT1 shortened the half-life of CDC6 mRNA (Fig. 6F, G). Taken together, these results demonstrated that the LCAT1-IGF2BP2 axis indeed regulates the stability of CDC6 mRNA, which is in a manner dependent on m^6^A modification.

## Discussion

LncRNAs have been reported to participate in tumorigenesis and tumor progression of a variety of cancers, including lung cancer [40–44]. However, only a few lncRNAs have been thoroughly characterized in lung cancer. In our previous study, we identified and characterized a novel lncRNA, LCAT1, in lung cancer [19] where LCAT1 is upregulated and associated with poor prognosis in lung cancer patients. LCAT1 was found to act as a ceRNA to regulate RAC1 function by sponging miR-4715-5p. However, this only partially explained the function of LCAT1 as an oncogenic lncRNA in lung cancer, and the additional underlying mechanism of LCAT1 action remained to be elusive.

In addition to acting as a miRNA sponge to regulate the function of target genes, lncRNAs more generally operate via physical interactions with regulatory proteins to affect their biological functions [45–47]. In the present study, we pursued further the mechanism of LCAT1 action by employing RNA pull-down, MS and RIP assays to identify potential binding proteins of LCAT1, of which IGF2BP2 was found as one of the major candidates.

IGF2BP2 is a member of the insulin-like growth factor 2 (IGF2) mRNA-binding proteins family and participates in post-transcriptional gene regulation [48, 49]. IGF2BP2 consists of two RRM domains and four KH domains, responsible for its RNA-binding function. To define how LCAT1 interacted with IGF2BP2, we mapped the KH domain of IGF2BP2 as the LCAT1 binding region via a series of deletion mutants. Interestingly, we further found that LCAT1 can maintain the protein stability of IGF2BP2 by preventing its degradation by autolysosomes. However, the mechanism by which IGF2BP2 binds to LCAT1 through its KH domain to prevent it from being degraded by autolysates is unclear and requires further investigation.

Accumulating lines of evidence show IGF2BP2 as upregulated in multiple human cancers and suggest its important role in tumorigenesis and tumor progression [50, 51]. For instance, IGF2BP2 promotes colorectal cancer cell proliferation and survival via disturbing RAF-1 degradation by miR-195 [52]. Several miRNAs, such as miR-320b and miR-485-5p, inhibit the progression of lung cancer by targeting IGF2BP2 [32, 53]. Indeed, in our study, IGF2BP2 was upregulated in lung tumor tissues, and its expression was highly predictive of poor prognosis for lung cancer patients. Through both loss-of-function (siRNA-based knockdown) and gain-of-function (ectopic overexpression) assays, we found that IGF2BP2 is a positive regulator of proliferation, colonic survival, and migration in lung cancer cells both in in vitro and in vivo.

What is the underlying mechanism of IGF2BP2 acting as an oncogenic protein? Importantly, the IGF2BP family members were well-known to function as m^6^A readers, which recognize the m^6^A-modified target RNAs to regulate their stability or translation. The m^6^A is the most abundant RNA modification and its biological function depends on an m^6^A reader protein. For instance, the m6A reader IGF2BP3 recognizes and binds to the m6A site on HDGF mRNA, thereby enhancing the stability of HDGF mRNA in gastric cancer [54]. Likewise, IGF2BP2, an important m^6^A reader protein, may affect many of its downstream target genes. However, which target genes primarily affect lung cancer remained to be determined.

To further elucidate the mechanism of LCAT1-IGF2BP2 action in our experimental setting, we went on and identified a handful of downstream targets of IGF2BP2. We followed up with one of these candidates, CDC6 (cell division cycle 6), a critical cell cycle regulator that plays an essential role in the maintenance of genome stability [55] via recruiting cdt1-MCM2-7 complexes to the origin of replication [56]. CDC6 is also known as an oncogenic target [57]. Previous studies have shown CDC6 as dysregulated in many cancers, such as pancreatic cancer [58], mantle cell lymphoma [59], and hepatocellular carcinoma [60]. However, the biological role of CDC6 in lung cancer remains to be further elucidated. To this end, we thoroughly characterized CDC6 as a new m^6^A target of the LCAT1/IGF2BP2 complex, which promotes CDC6 expression in lung cancer cells. Specifically, using MeRIP-qPCR assays, we found that CDC6 mRNA was modified by N^6^-methyladenosine, which was then recognized by the m^6^A reader protein IGF2BP2. The recognition between IGF2BP2 and CDC6 mRNA could be abolished by knocking out the m^6^A-modified core methylase METTL3, suggesting that the recognition is m^6^A-dependent. We further demonstrated that the LCAT/IGF2BP2 complex could enhance the stability of CDC6 mRNA, thereby leading to the upregulation of CDC6 protein expression. Importantly, CDC6 is upregulated in lung cancer and its higher expression is associated with a worse outcome for lung cancer patients. Biologically, CDC6 promoted cell proliferation, colony formation, and migration of lung cancer cells, and forced expression of CDC6 can overcome the cellular effects caused by the blockage of the LCAT1-IGF2BP2 axis, implicating that the effect of LCAT1-IGF2BP2 is at least in part mediated by downstream CDC6.

In summary, our study fits a novel working model as follows: LCAT1 binds and stabilizes an m^6^A reader protein IGF2BP2 by preventing its lysosomal degradation. Stabilized IGF2BP2 then upregulates the expression of CDC6 in an m^6^A-dependent manner. CDC6 then acts as an oncogenic protein to promote the proliferation, survival, and migration of lung cancer cells (Fig. 6H). Thus, the oncogenic LCAT1-IGF2BP2-CDC6 axis appears to be a potential therapeutic target for lung cancer and may serve as a promising prognostic biomarker, given its association with poor patient survival.

## Supplementary information

Supplementary Table S1-S4 and Figure S1-S6

Reproducibility checklist

Original Data File

all authors agree to added Jiayi Ren as a new author

## Data Availability

All data generated during this study are included in this published article and its supplementary files.
