# Supplementary material for: LCAT1 is an oncogenic LncRNA by stabilizing the IGF2BP2-CDC6 axis
Source: Cell Death Dis. 2022 Oct 18;13(10):877. doi: 10.1038/s41419-022-05316-4 (PMC9579176; doi:10.1038/s41419-022-05316-4)
Supplement: Supplementary file 1 — Supplementary Table S1-S4 and Figure S1-S6 [file 41419_2022_5316_MOESM1_ESM.pdf]

LCAT1 is an oncogenic LncRNA by stabilizing the IGF2BP2-CDC6 axis

Juze Yang, Xinyi Qian, Qiongzi Qiu, Lingling Xu, Meidie Pan, Jia Li, Jiayi Ren, Bingjian Lu, Ting Qiu, Enguo Chen, Kejing Ying, Honghe Zhang, Yan Lu, Pengyuan Liu

Supplementary data

Supplementary Table 1. List of reagents and antibodies used in this study

**Name**	**Manufacturer**	**Cat. no.**
Cycloheximide	MedChem Express	HY-12320
bafilomycin A1	MedChem Express	HY-100558
NH_4Cl	Sangon Biotech	A100621
MG-132	Selleck Chemicals	S2619
Rapamycin	Selleck Chemicals	S1035
AZD8055	Selleck Chemicals	S1555
Anti-FLAG® M2 affinity gel	Sigma-Aldrich	A2220
EBSS	Thermo Fisher Scientific	14155063
3-methyladenine	Sigma-Aldrich	M9281
GAPDH	Proteintech Group	60004-1-Ig
CDC6	Cell Signaling Technology	3387
IGF2BP2	Santa Cruz Biotechnology	sc-377014
METTL3	Proteintech Group	15073-1-AP
ULK1	Cell Signaling Technology	8054
m6A	Synaptic Systems	202011

Supplementary Table 2. Sequences of siRNAs used in this study.

**Oligo name**	**Sequence**
LCAT1 siRNA 1#	AAUCUCCCAUUGACUGAGC
LCAT1 siRNA 2#	AAGUUGGCUGUAACUCUGG
IGF2BP2 siRNA 1#	UAAGUUCUGCAGUUCGUUC
IGF2BP2 siRNA 2#	AAUUUCCCUGAUCUUGCGC
IGF2BP2 siRNA 3#	UGUUAUCUUGGUCCCUGUU
CDC6 siRNA 1#	AAAGGUAAAGGCUUCCAG
CDC6 siRNA 2#	AUGUGAAUAAGACCAACCC
CDC6 siRNA 3#	AUCUUGUGCUCCUUCUUGG
ULK1 siRNA 1#	AGAAUUAGCCAUUUCCUGGAA
ULK1 siRNA 2#	UAGUGCUGGGACAUGAUGACC
ULK1 siRNA 3#	UUAAGGAGCAGGUCAGUGAGG

Supplementary Table 3. Quality control metrics of RNA-seq libraries

**Sample ID**	**Yield (Gb)**	**#Reads**	**%of>=Q30 Bases (PF)**	**Mean Quality Score (PF)**	**%Of Mapping Rate**
siCrtl1	8.77	62,804,374	96.26	37.77	89.80
siCrtl2	9.41	67,376,556	96.47	37.85	90.50
siCrtl3	8.99	64,354,262	96.45	37.84	90.70
siIGF2BP2 1#-1	9.48	67,862,530	96.67	37.92	88.90
siIGF2BP2 1#-2	9.11	65,210,096	96.45	37.84	87.00
siIGF2BP2 1#-3	8.62	61,689,044	96.68	37.90	87.50
siIGF2BP2 2#-1	9.22	65,987,158	96.63	37.90	89.20
siIGF2BP2 2#-2	9.80	70,165,158	96.55	37.86	88.20
siIGF2BP2 2#-3	9.18	65,722,210	96.41	37.82	88.20

Supplementary Table 4. Proteins identified by mass spectrometry analysis of RNA pull-down fractions.

**Protein name**	**Unique peptides**		**Mol. Weight [kDa]**
	**Antisense**	**Sense**	
G3BP1	5	12	52.164
NONO	8	12	54.231
HNRPK	5	10	50.976
U2AF2	3	6	53.5
KHDR1	1	3	48.227
**IGF2BP2**	**0**	**4**	**66.121**
LBR	0	3	70.702
FA98A	0	2	55.4
PNKP	0	2	57.076

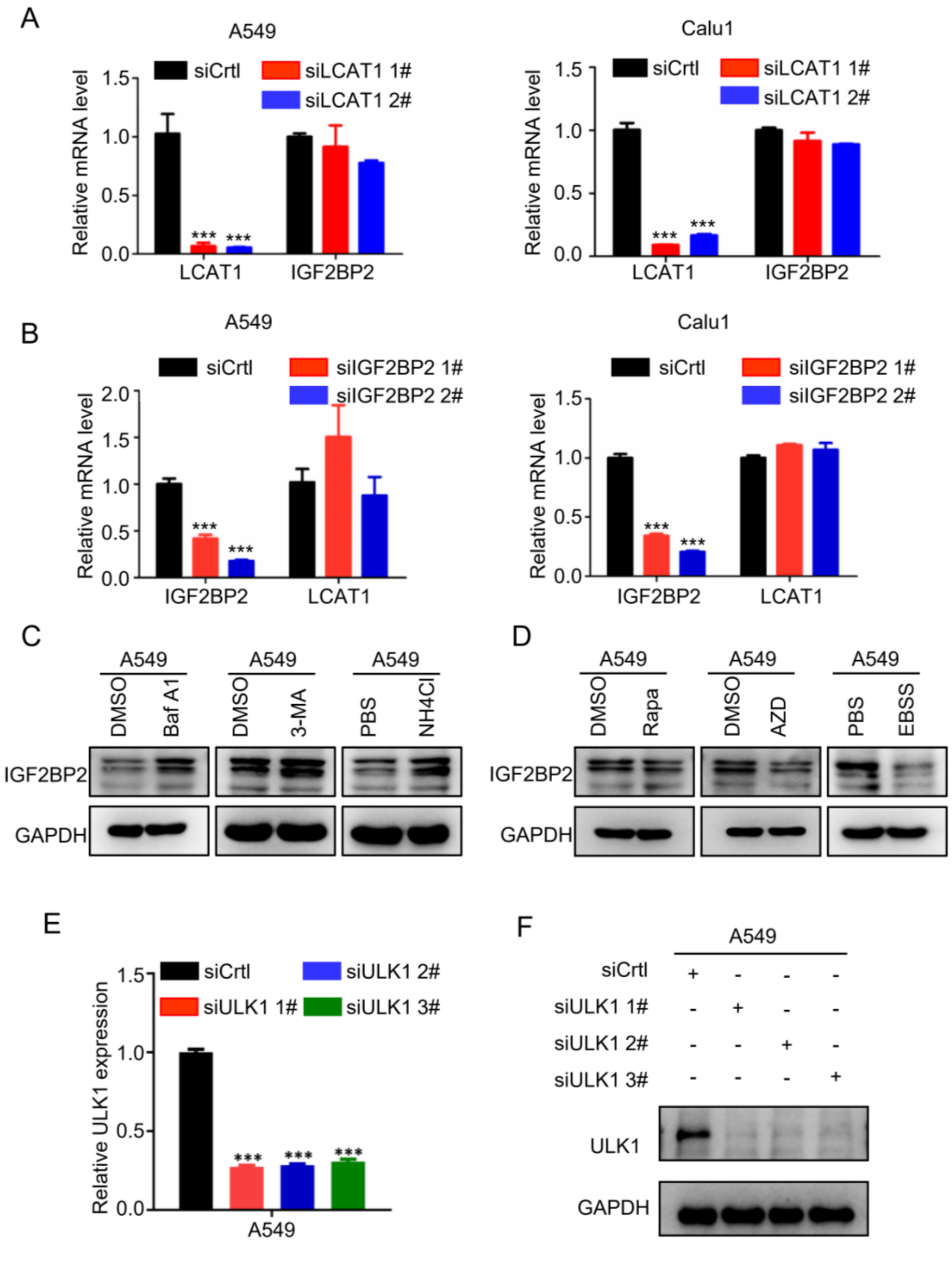

Supplementary Fig. 1 (**A**) LCAT1 and IGF2BP2 expression was measured by qPCR in LCAT1 knockdown cells. (**B**) LCAT1 and IGF2BP2 expression was measured by qPCR in IGF2BP2 knockdown cells. (**C**) Immunoblotting of IGF2BP2 in lung cancer cells with or without treatment of Bafilomycin A1, 3-MA or NH4Cl. Cell lysates were analyzed by western blotting with GAPDH as a loading control. (**D**) Immunoblotting of IGF2BP2 in lung cancer cells with or without treatment of Rapamycin, AZD8055 or EBSS. (**E, F**) qPCR and immunoblotting were employed to detect the ULK1 expression in lung cancer cells transfected with siRNA or negative controls. Data are presented as mean ± SD. *P < 0.05; **P < 0.01; ***P < 0.001.

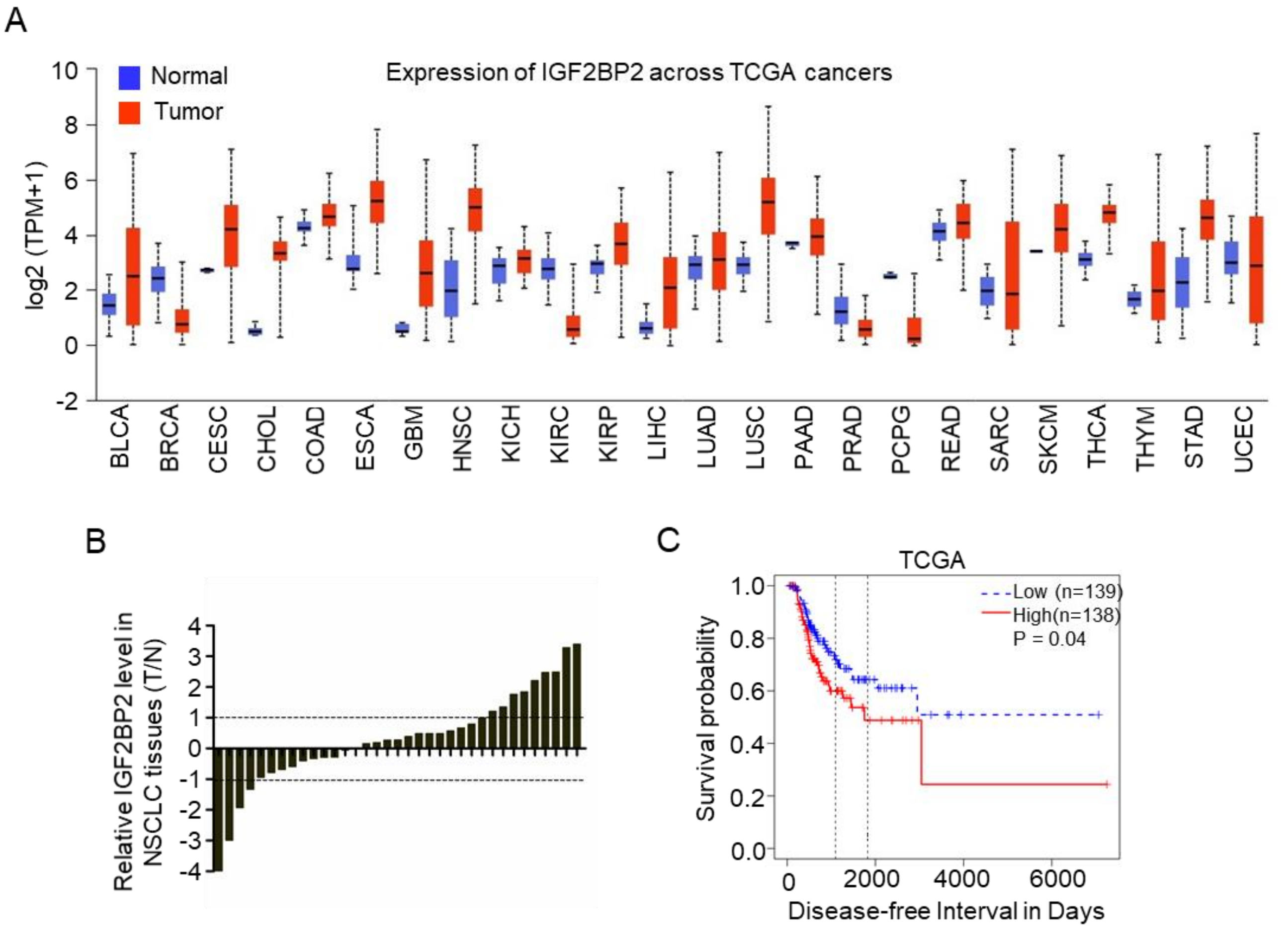

Supplementary Fig. 2 (**A**) The mRNA expression of IGF2BP2 in different cancer types of the TCGA. (**B**) qPCR was performed to detect IGF2BP2 expression in 35 new lung cancer tissues and matched adjacent non-tumor tissues. (**C**) Kaplan-Meier curves of disease-free survival time based on IGF2BP2 expression of lung cancer patients of the TCGA.

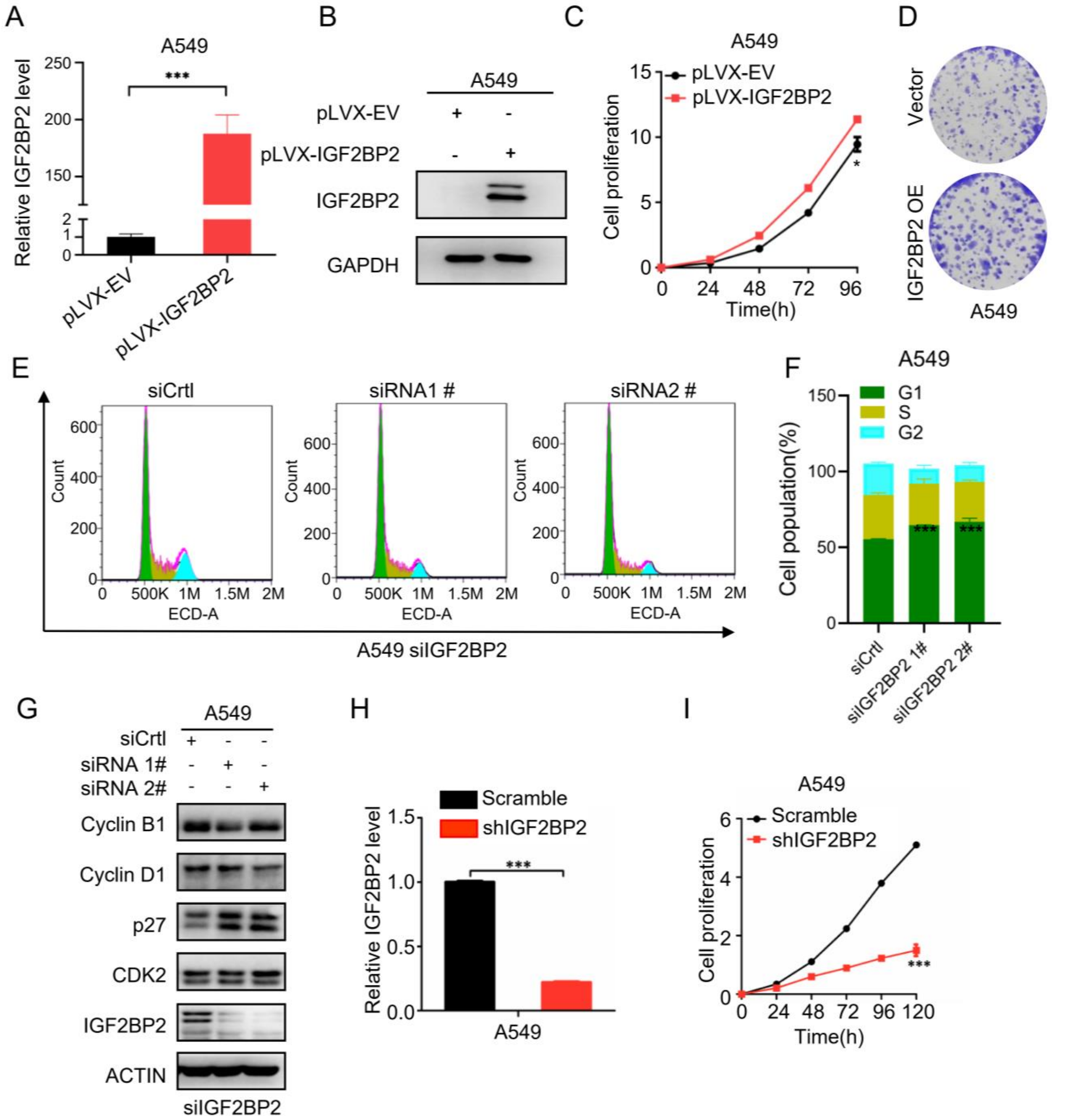

Supplementary Fig. 3 (**A, B**) IGF2BP2 mRNA and protein were measured in IGF2BP2 overexpressing cells. (**C, D**) CCK8 assay and colony formation assay were used to measure the growth of A549 cells after overexpression of IGF2BP2. (**E, F**) PI staining and flow cytometry were used to examine the G0/G1 cell cycle transition in IGF3BP2-knockdown A549 cells. (G) Western blots were conducted to detect cell cycle-related proteins. (**H**) qPCR was performed to detect IGF2BP2 expression in shCrtl or shIGF2BP2 cells. (**I**) CCK8 assay was used to evaluate the influence of IGF2BP2 knockdown on cell proliferation. Student's two-sided t tests were used. *P < 0.05, ** P < 0.01, ***P < 0.001.

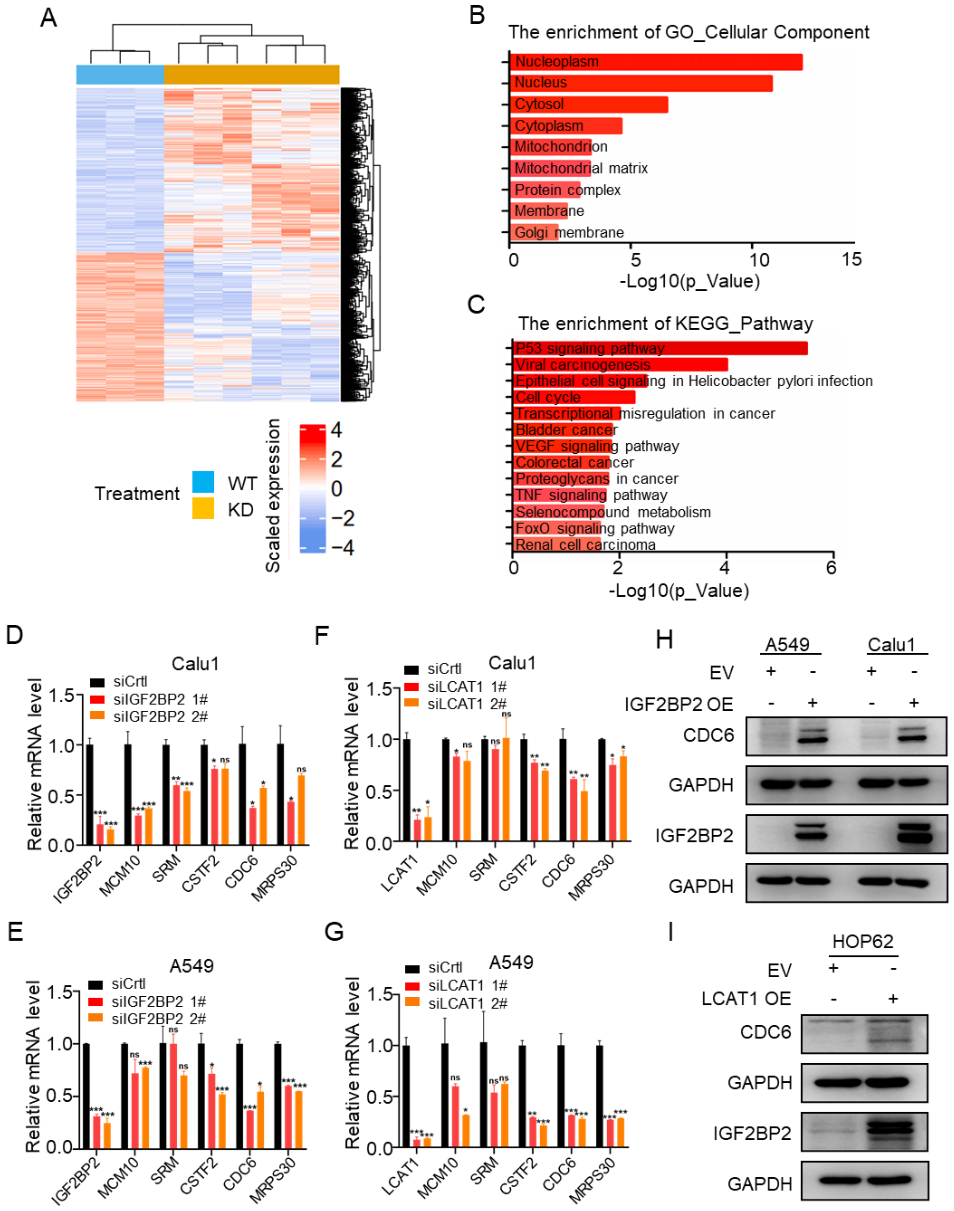

Supplementary Fig. 4 (**A**) Heatmap of genes altered in Calu1 cells with IGF2BP2 knockdown (two siRNAs). (**B**) Gene ontology (GO) analysis of genes altered in Calu1 cells with IGF2BP2 knockdown. (**C**) KEGG pathway analysis of genes altered in Calu1 cells with IGF2BP2 knockdown. (**D, E**) Relative mRNA expression of IGF2BP2 and LCAT1/IGF2BP2 target genes, including MCM10, SRM, CSTF2, CDC6, MRPS30 in Calu1 and A549 cells, were examined by qPCR upon IGF2BP2 knockdown. (**F, G**) Relative mRNA expression of LCAT1 and LCAT1/IGF2BP2 target genes were examined by qPCR upon LCAT1 knockdown. (**H**) Immunoblotting of CDC6 in IGF2BP2 overexpressing cells. (**I**) CDC6 expression was detected in LCAT1 overexpressed cells. Statistics were performed using Student's two-sided t test. *$P < 0.05$, ** $P < 0.01$, ***$P < 0.001$.

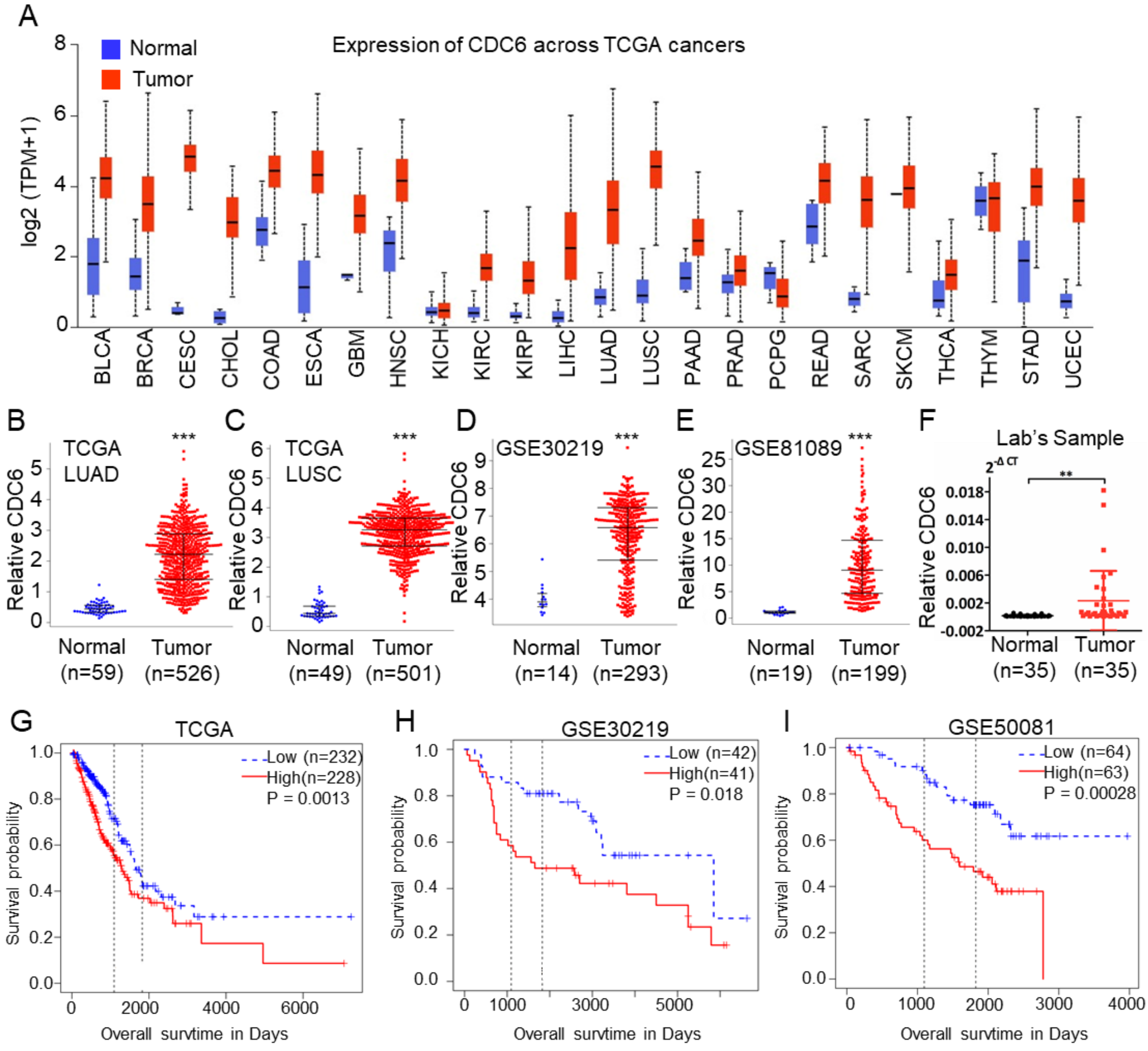

Supplementary Fig. 5 (**A**) The mRNA expression of CDC6 in different cancer types of the TCGA. (**B-E**) The relative expression level of CDC6 in TCGA-LUAD, TCGA-LUSC, GSE30219 and GSE81089, respectively. (**F**) qPCR was performed to detect CDC6 expression in 35 fresh NSCLC tissues and matched adjacent non-tumor tissues. (**G-I**) Kaplan-Meier curves of overall survival of lung cancer patients based on CDC6 mRNA expression. Data are presented as mean ± SD. $^{*}P < 0.05$; $^{**}P < 0.01$; $^{***}P < 0.001$.

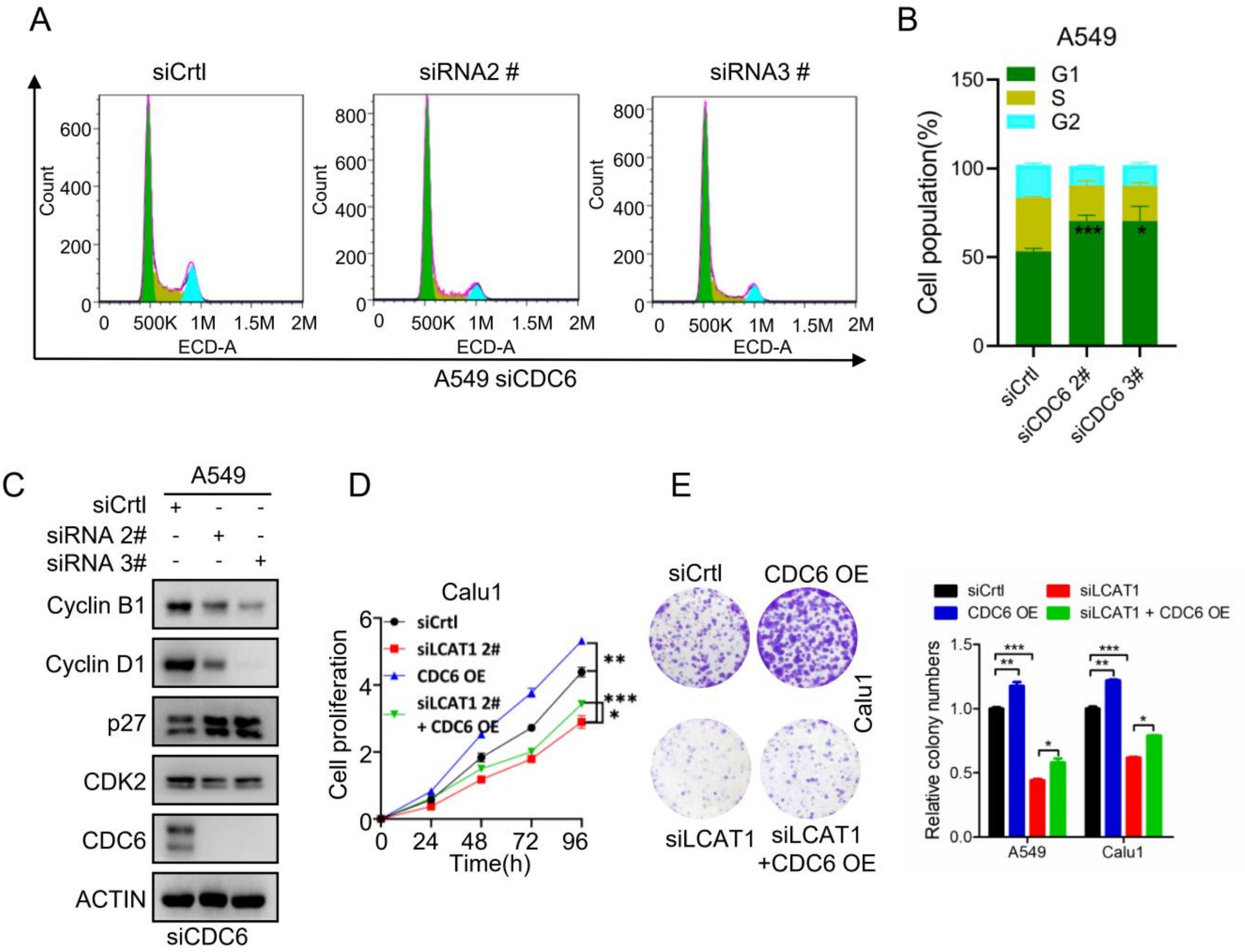

Supplementary Fig. 6 (**A, B**) PI staining and flow cytometry were used to examine the G0/G1 cell cycle transition was examined in CDC6-knockdwon A549 cells. (**C**) Western blots were conducted to detect cell cycle-related proteins. (**D, E**) Growth curve and colony formation assays were performed in Calu1 cells co-transfected with siLCAT1 and CDC6 overexpression vectors. *P < 0.05, ** P < 0.01, ***P < 0.001.
