# Supplementary material for: LCAT1 is an oncogenic LncRNA by stabilizing the IGF2BP2-CDC6 axis
Source: Cell Death Dis. 2022 Oct 18;13(10):877. doi: 10.1038/s41419-022-05316-4 (PMC9579176; doi:10.1038/s41419-022-05316-4)
Supplement: Supplementary file 3 — Original Data File [file 41419_2022_5316_MOESM3_ESM.pdf]

Fig 1C

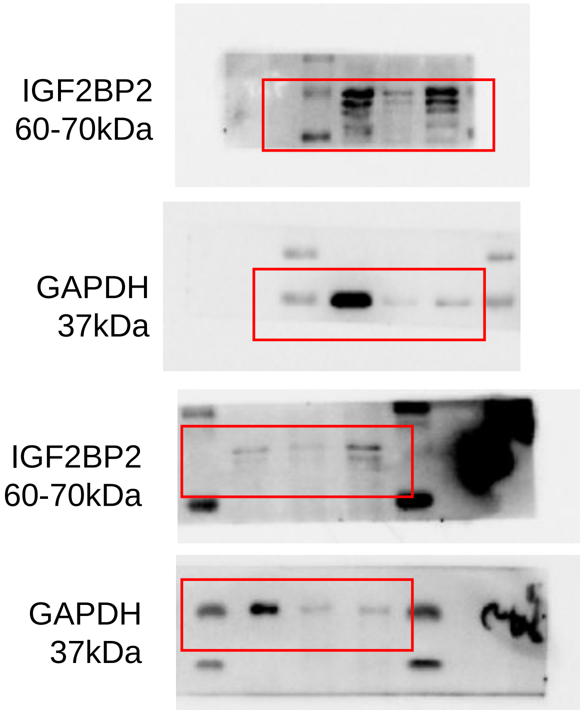

Fig 1D

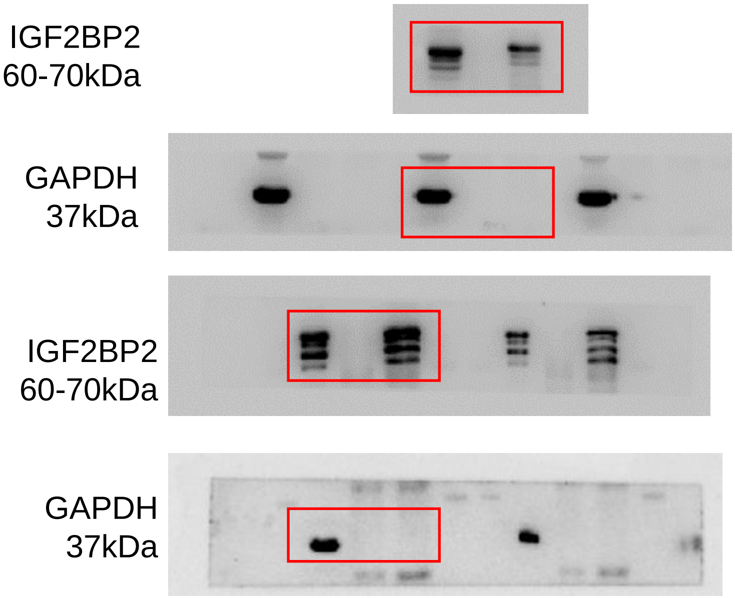

Fig 1G

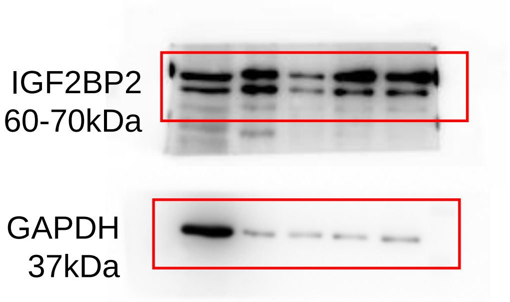

Fig 2A

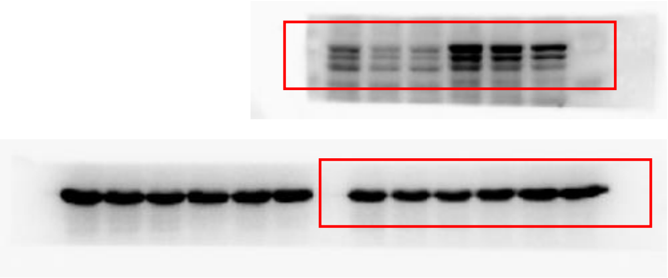

Fig 2B

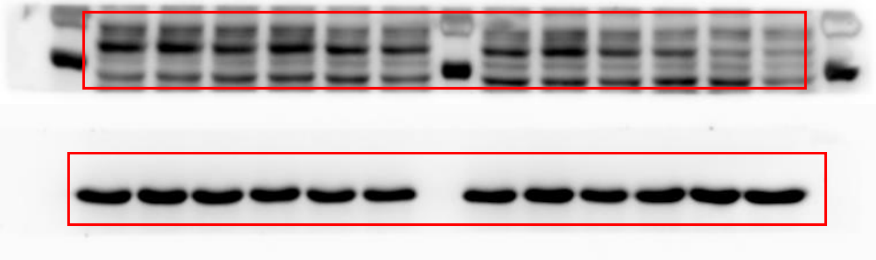

Fig 2D

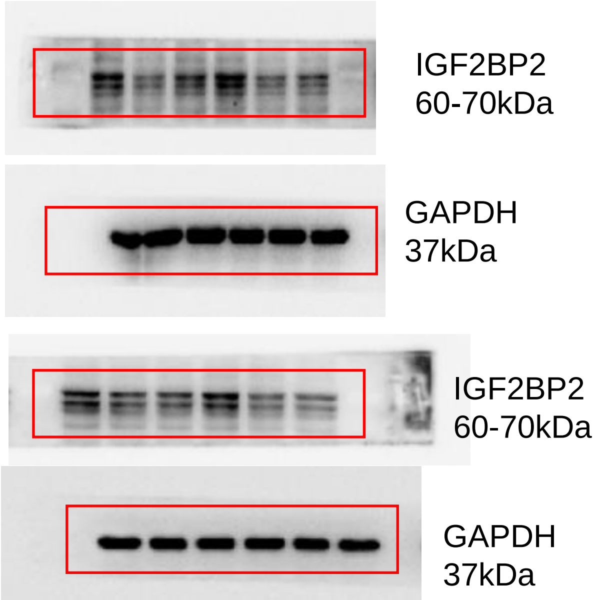

Fig 2E

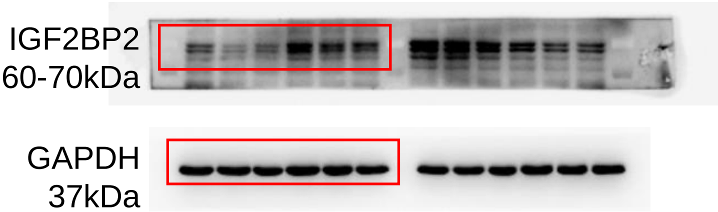

Fig 2F

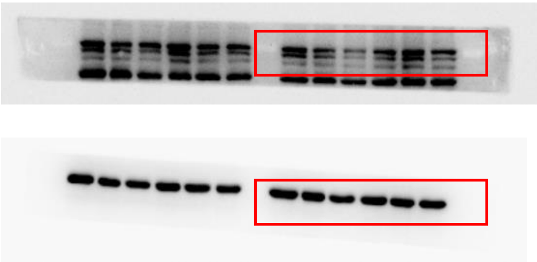

Fig 3B

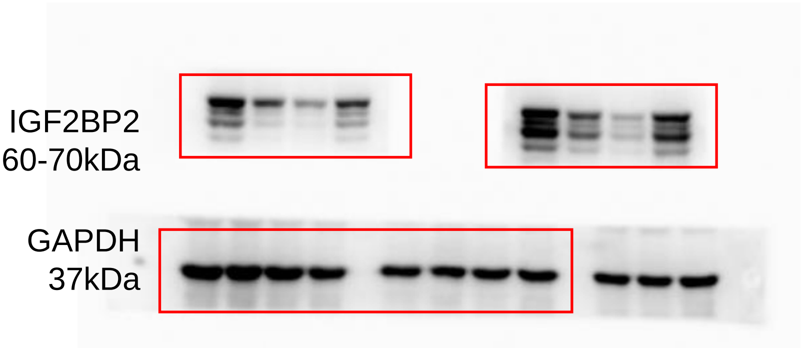

Fig 4J and 4H

CDC6
62kDa

GAPDH
37kDa

IGF2BP2
60-70kDa

GAPDH
37kDa

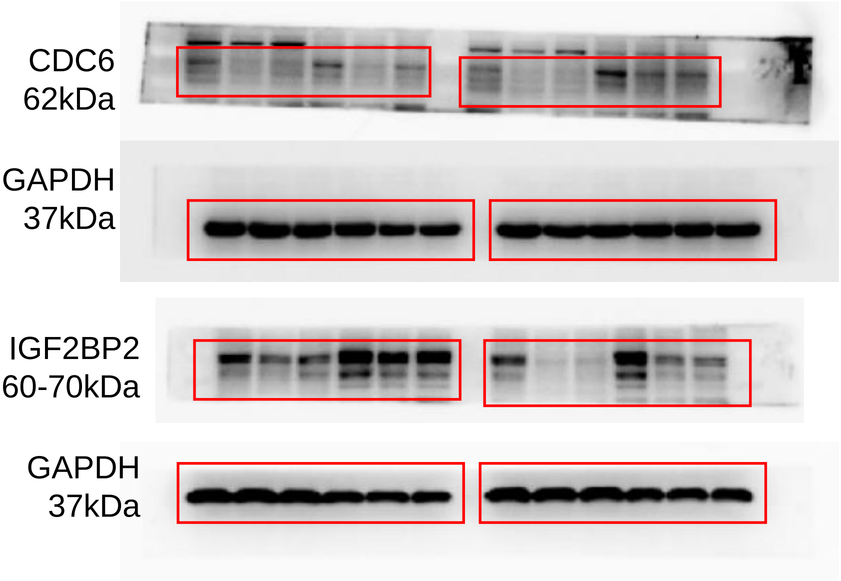

Fig 4L

CDC6
62kDa

GAPDH
37kDa

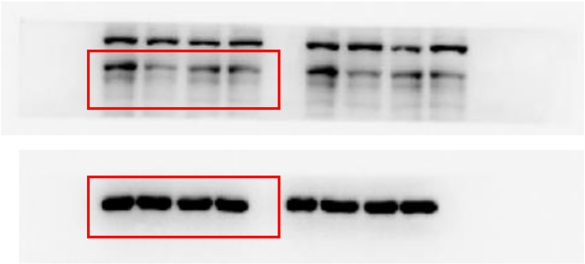

Fig 5B

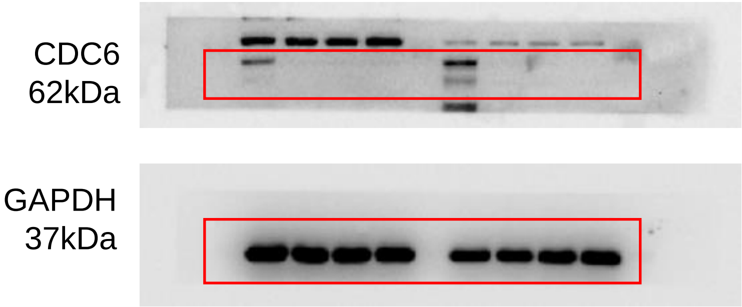

Fig 6C

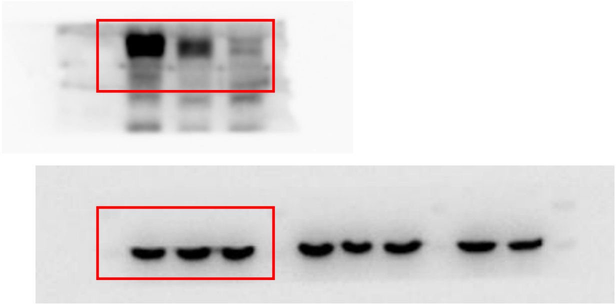

METTL3
70kDa

GAPDH
37kDa

Fig S1C

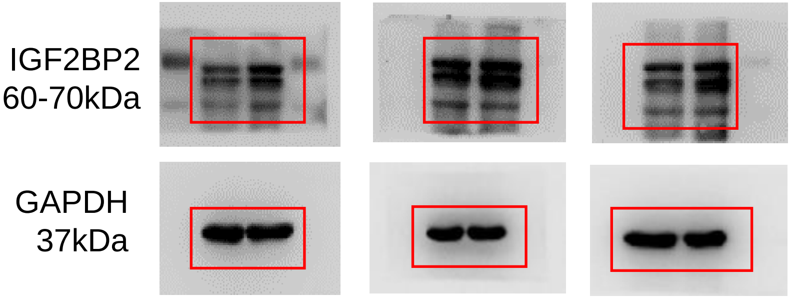

Fig S1D

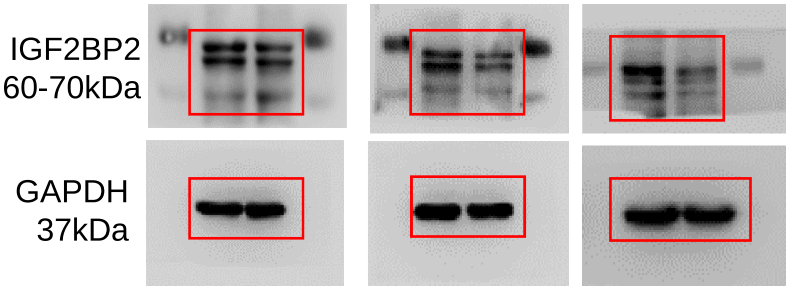

Fig S1F

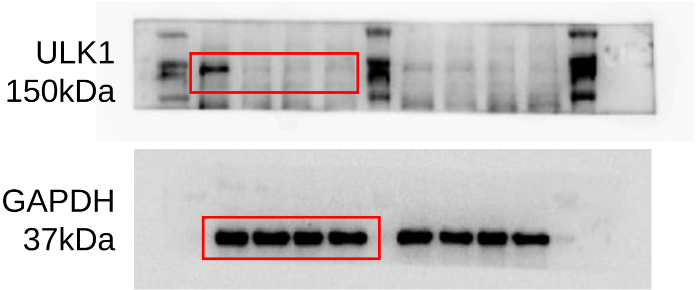

Fig S3B

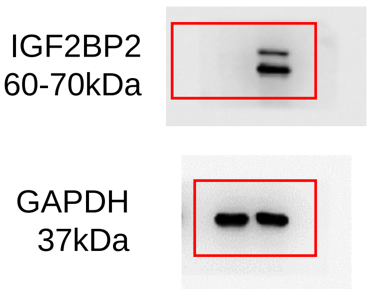

Fig S3G

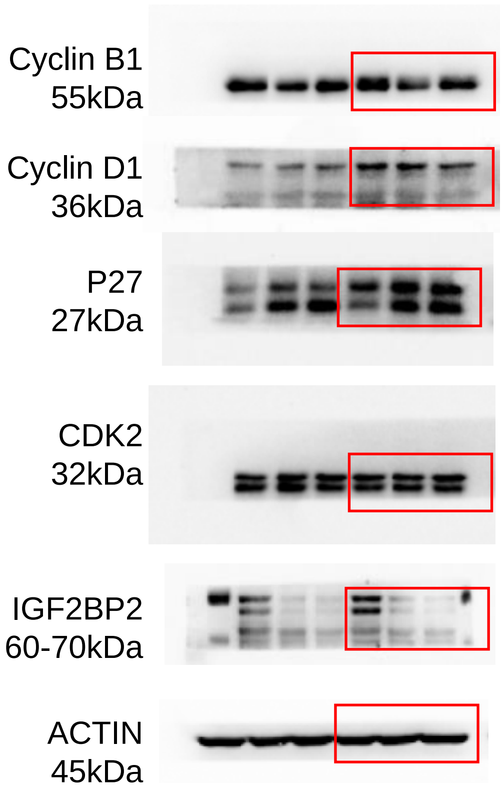

Fig S4H

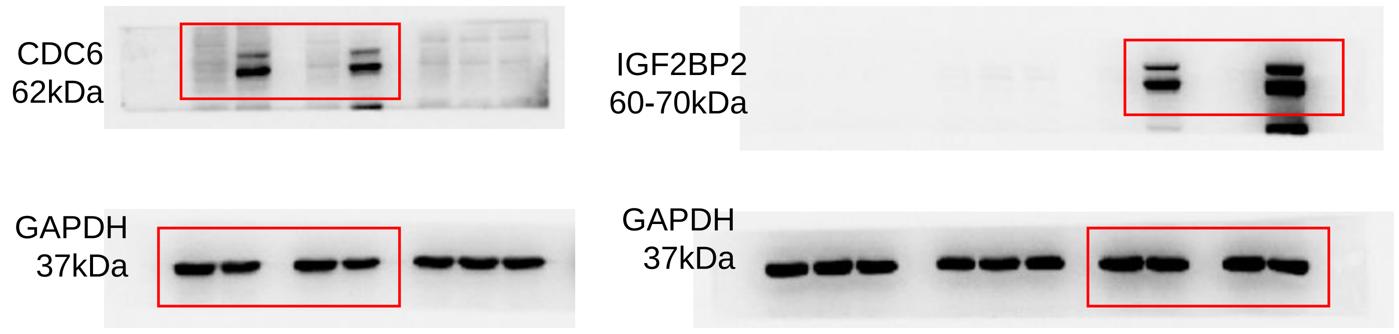

Fig S4I

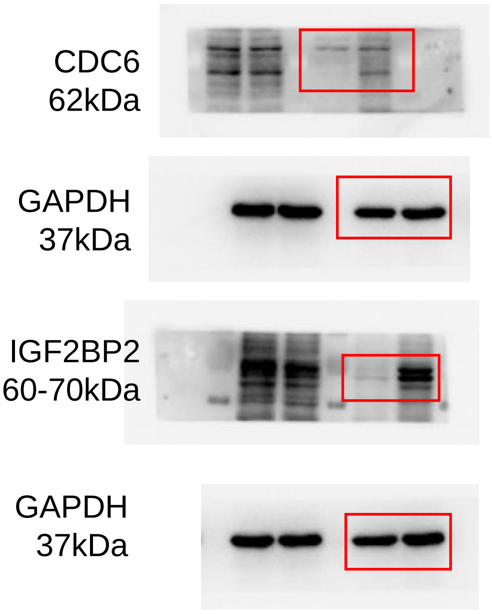

Fig S6C

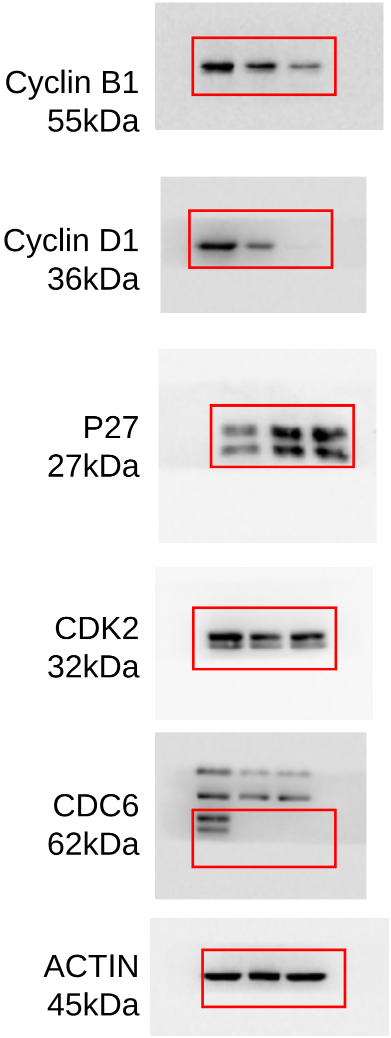

Cyclin B1
55kDa
Cyclin D1
36kDa
P27
27kDa
CDK2
32kDa
CDC6
62kDa
ACTIN
45kDa
