# Supplementary material for: LCAT1 is an oncogenic LncRNA by stabilizing the IGF2BP2-CDC6 axis
Source: Cell Death Dis. 2022 Oct 18;13(10):877. doi: 10.1038/s41419-022-05316-4 (PMC9579176; doi:10.1038/s41419-022-05316-4)
Supplement: Supplementary file 4 — all authors agree to added Jiayi Ren as a new author [file 41419_2022_5316_MOESM4_ESM.pdf]

Re: RE: A new author was added in CDDIS-22-2707RR

2022-09-28 19:15:09

发件人： "杨举泽" <21518450@zju.edu.cn>

收件人： pyliu@zju.edu.cn

抄 送： 21718673@zju.edu.cn 3110102477@zju.edu.cn 389088167@qq.com 3180102403@zju.edu.cn jial_jacky@163.com .. [↓还有7个联系人]

I agree
Juze Yang

2022-09-28 18:00:18pyliu@zju.edu.cn写道：

> Dear All:
>
> Jiayi Ren has been added to the author list for her contribution to data analysis in a revised manuscript submitted to *Cell Death & Disease* entitled "LCAT1 is an oncogenic LncRNA by stabilizing IGF2BP2-CDC6 axis". If you agree to this change, please reply to this email "I agree" and enter your name.
>
> Thanks for your contribution to this paper!
>
> Pengyuan Liu

回复：A new author was added in CDDIS-22-2707RR

2022-09-28 18:13:38

发件人： "21718673@zju.edu.cn" <21718673@zju.edu.cn>

收件人： "pyliu@zju.edu.cn" <pyliu@zju.edu.cn>

I agree.
Xinyi Qian

发自我的手机

——— 原始邮件 ———

发件人： pyliu@zju.edu.cn

日期： 2022年9月28日周三 傍晚6:00

收件人： 21518450@zju.edu.cn, 21718673@zju.edu.cn, 3110102477@zju.edu.cn, 389088167@qq.com, 3180102403@zju.edu.cn, jial_jacky@163.com, lbj@zju.edu.cn, phoebe837@163.com, 3195024@zju.edu.cn, 3197061@zju.edu.cn, honghezhang@zju.edu.cn, 陆燕 <yanlu76@zju.edu.cn>, 刘鹏渊 <pyliu@zju.edu.cn>, renjy1998@zju.edu.cn

主 题： RE: A new author was added in CDDIS-22-2707RR

Dear All:

Jiayi Ren has been added to the author list for her contribution to data analysis in a revised manuscript submitted to *Cell Death & Disease* entitled "LCAT1 is an oncogenic LncRNA by stabilizing IGF2BP2-CDC6 axis". If you agree to this change, please reply to this email "I agree" and enter your name.

Thanks for your contribution to this paper!

Pengyuan Liu

RE: A new author was added in CDDIS-22-2707RR

发件人： "3110102477" <3110102477@zju.edu.cn>

收件人： pyliu@zju.edu.cn

2022-09-29 08:43:47

I agree.

Qiongzi Qiu

-------- Original message --------
From: pyliu@zju.edu.cn
Date: 9/28/22 18:00 (GMT+08:00)
To: 21518450@zju.edu.cn, 21718673@zju.edu.cn, 3110102477@zju.edu.cn, 389088167@qq.com, 3180102403@zju.edu.cn, jial_jacky@163.com, lbj@zju.edu.cn, phoebe837@163.com, 3195024@zju.edu.cn, 3197061@zju.edu.cn, honghezhang@zju.edu.cn, 陆燕 <yanlu76@zju.edu.cn>, 刘鹏渊 <pyliu@zju.edu.cn>, renjy1998@zju.edu.cn
Subject: RE: A new author was added in CDDIS-22-2707RR

Dear All:

Jiayi Ren has been added to the author list for her contribution to data analysis in a revised manuscript submitted to *Cell Death & Disease* entitled "LCAT1 is an oncogenic LncRNA by stabilizing IGF2BP2-CDC6 axis". If you agree to this change, please reply to this email "I agree" and enter your name.

Thanks for your contribution to this paper!

Pengyuan Liu

Re: RE: A new author was added in CDDIS-22-2707RR

发件人： "Lingling Xu" <389088167@qq.com>

收件人： "pyliu" <pyliu@zju.edu.cn>

2022-09-28 18:32:41

I agree, Lingling Xu.

---Original---

From: "pyliu"<pyliu@zju.edu.cn>
Date: Wed, Sep 28, 2022 18:00 PM
To: "21518450"<21518450@zju.edu.cn>;"21718673"<21718673@zju.edu.cn>;"3110102477"<3110102477@zju.edu.cn>;"389088167"<389088167@qq.com>;"3180102403"<3180102403@zju.edu.cn>;"jial_jacky"<jial_jacky@163.com>;"lbj"<lbj@zju.edu.cn>;"phoebe837"<phoebe837@163.com>;"3195024"<3195024@zju.edu.cn>;"3197061"<3197061@zju.edu.cn>;"honghezhang"<honghezhang@zju.edu.cn>;"陆燕"<yanlu76@zju.edu.cn>;"刘鹏渊"<pyliu@zju.edu.cn>;"renjy1998"<renjy1998@zju.edu.cn>;
Subject: RE: A new author was added in CDDIS-22-2707RR

Dear All:

Jiayi Ren has been added to the author list for her contribution to data analysis in a revised manuscript submitted to *Cell Death & Disease* entitled "LCAT1 is an oncogenic LncRNA by stabilizing IGF2BP2-CDC6 axis". If you agree to this change, please reply to this email "I agree" and enter your name.

Thanks for your contribution to this paper!

Pengyuan Liu

Re: RE: A new author was added in CDDIS-22-2707RR

2022-09-28 22:09:50

发件人： 3180102403@zju.edu.cn

收件人： pyliu@zju.edu.cn

I agree.

Meidie Pan

——原始邮件——

发件人:pyliu@zju.edu.cn

发送时间:2022-09-28 18:00:18 (星期三)

收件人: 21518450@zju.edu.cn, 21718673@zju.edu.cn, 3110102477@zju.edu.cn, 389088167@qq.com, 3180102403@zju.edu.cn, jial_jacky@163.com, lbj@zju.edu.cn, phoebe837@163.com, 3195024@zju.edu.cn, 3197061@zju.edu.cn, honghezhang@zju.edu.cn, "陆燕" <yanlu76@zju.edu.cn>, "刘鹏渊" <pyliu@zju.edu.cn>, renjy1998@zju.edu.cn

抄送:

主题: RE: A new author was added in CDDIS-22-2707RR

Dear All:

Jiayi Ren has been added to the author list for her contribution to data analysis in a revised manuscript submitted to *Cell Death & Disease* entitled "LCAT1 is an oncogenic LncRNA by stabilizing IGF2BP2-CDC6 axis". If you agree to this change, please reply to this email "I agree" and enter your name.

Thanks for your contribution to this paper!

Pengyuan Liu

Re:RE: A new author was added in CDDIS-22-2707RR

2022-09-28 20:24:02

发件人： "lj" <jial_jacky@163.com>

收件人： pyliu@zju.edu.cn

I agree.

Li Jia

At 2022-09-28 18:00:18, pyliu@zju.edu.cn wrote:

Dear All:

Jiayi Ren has been added to the author list for her contribution to data analysis in a revised manuscript submitted to *Cell Death & Disease* entitled "LCAT1 is an oncogenic LncRNA by stabilizing IGF2BP2-CDC6 axis". If you agree to this change, please reply to this email "I agree" and enter your name.

Thanks for your contribution to this paper!

Pengyuan Liu

Re: RE: A new author was added in CDDIS-22-2707RR

2022-09-28 19:15:36

发件人： "任珈仪" <12118596@zju.edu.cn>

收件人： pyliu@zju.edu.cn

I agree

Jiayi Ren

——原始邮件——
发件人: pyliu@zju.edu.cn
发送时间: 2022-09-28 18:00:18 (星期三)
收件人: 21518450@zju.edu.cn, 21718673@zju.edu.cn, 3110102477@zju.edu.cn, 389088167@qq.com, 3180102403@zju.edu.cn, jial_jacky@163.com, lbj@zju.edu.cn, phoebe837@163.com, 3195024@zju.edu.cn, 3197061@zju.edu.cn, honghezhang@zju.edu.cn, "陆燕" <yanlu76@zju.edu.cn>, "刘鹏渊" <pyliu@zju.edu.cn>, renjy1998@zju.edu.cn
抄送:
主题: RE: A new author was added in CDDIS-22-2707RR

Dear All:

Jiayi Ren has been added to the author list for her contribution to data analysis in a revised manuscript submitted to *Cell Death & Disease* entitled "LCAT1 is an oncogenic LncRNA by stabilizing IGF2BP2-CDC6 axis". If you agree to this change, please reply to this email "I agree" and enter your name.

Thanks for your contribution to this paper!

Pengyuan Liu

Re: RE: A new author was added in CDDIS-22-2707RR

2022-09-28 22:59:0

发件人： lbj@zju.edu.cn

收件人： pyliu@zju.edu.cn

Dear Pengyuan,

I agree.

Bingjian Lu

——原始邮件——
发件人: pyliu@zju.edu.cn
发送时间: 2022-09-28 18:00:18 (星期三)
收件人: 21518450@zju.edu.cn, 21718673@zju.edu.cn, 3110102477@zju.edu.cn, 389088167@qq.com, 3180102403@zju.edu.cn, jial_jacky@163.com, lbj@zju.edu.cn, phoebe837@163.com, 3195024@zju.edu.cn, 3197061@zju.edu.cn, honghezhang@zju.edu.cn, "陆燕" <yanlu76@zju.edu.cn>, "刘鹏渊" <pyliu@zju.edu.cn>, renjy1998@zju.edu.cn
抄送:
主题: RE: A new author was added in CDDIS-22-2707RR

Dear All:

Jiayi Ren has been added to the author list for her contribution to data analysis in a revised manuscript submitted to *Cell Death & Disease* entitled "LCAT1 is an oncogenic LncRNA by stabilizing IGF2BP2-CDC6 axis". If you agree to this change, please reply to this email "I agree" and enter your name.

Thanks for your contribution to this paper!

Pengyuan Liu

Re:RE: A new author was added in CDDIS-22-2707RR

2022-09-28 20:46:31

发件人： "邱婷" <phoebe837@163.com>

收件人： pyliu@zju.edu.cn

I agree

At 2022-09-28 18:00:18, pyliu@zju.edu.cn wrote:

> Dear All:
>
> Jiayi Ren has been added to the author list for her contribution to data analysis in a revised manuscript submitted to *Cell Death & Disease* entitled "LCAT1 is an oncogenic LncRNA by stabilizing IGF2BP2-CDC6 axis". If you agree to this change, please reply to this email "I agree" and enter your name.
>
> Thanks for your contribution to this paper!
>
> Pengyuan Liu

Re: RE: A new author was added in CDDIS-22-2707RR

2022-09-28 22:09:5

发件人： 3195024@zju.edu.cn

收件人： pyliu@zju.edu.cn

I agree

enguo chen

> ——原始邮件——
> **发件人：**pyliu@zju.edu.cn
> **发送时间：**2022-09-28 18:00:18 (星期三)
> **收件人：** 21518450@zju.edu.cn, 21718673@zju.edu.cn, 3110102477@zju.edu.cn, 389088167@qq.com, 3180102403@zju.edu.cn, jial_jacky@163.com, lbj@zju.edu.cn, phoebe837@163.com, 3195024@zju.edu.cn, 3197061@zju.edu.cn, honghezhang@zju.edu.cn, "陆燕" <yanlu76@zju.edu.cn>, "刘鹏渊" <pyliu@zju.edu.cn>, renjy1998@zju.edu.cn
> **抄送：**
> **主题：** RE: A new author was added in CDDIS-22-2707RR
>
> Dear All:
>
> Jiayi Ren has been added to the author list for her contribution to data analysis in a revised manuscript submitted to *Cell Death & Disease* entitled "LCAT1 is an oncogenic LncRNA by stabilizing IGF2BP2-CDC6 axis". If you agree to this change, please reply to this email "I agree" and enter your name.
>
> Thanks for your contribution to this paper!
>
> Pengyuan Liu

Re:Fw: RE: A new author was added in CDDIS-22-2707RR

发起会议
2022-09-30 12:04:02

发件人： "3197061" <3197061@zju.edu.cn>

收件人： "pyliu" <pyliu@zju.edu.cn>

I agree .kejingYing

在 2022-09-30 11:36:47，pyliu@zju.edu.cn 写道：

>应老师：
>
>就是下面这个邮件，我是周三发的，麻烦您回复一下！
>
>谢谢！
>
>鹏渊
>
>
>
>———原始邮件———
>发件人:pyliu@zju.edu.cn
>发送时间:2022-09-28 18:00:18 (星期三)
>收件人：21518450@zju.edu.cn, 21718673@zju.edu.cn, 3110102477@zju.edu.cn, 389088167@qq.com, 3180102403@zju.edu.cn, jial_jacky@163.com, lbj@zju.edu.cn, phoebe837@163.com, 3195024@zju.edu.cn, 3197061@zju.edu.cn, honghezhang@zju.edu.cn, "陆燕" <yanlu76@zju.edu.cn>, "刘鹏渊" <pyliu@zju.edu.cn>, renjy1998@zju.edu.cn
>抄送：
>主题：RE: A new author was added in CDDIS-22-2707RR
>
>
>
>Dear All:
>
>
>
>
>Jiayi Ren has been added to the author list for her contribution to data analysis in a revised manuscript submitted to Cell Death & Disease entitled "LCAT1 is an oncogenic LncRNA by stabilizing IGF2BP2-CDC6 axis". If you agree to this change, please reply to this email "I agree" and enter your name.
>
>
>
>
>Thanks for your contribution to this paper!
>
>
>
>
>Pengyuan Liu

Re: RE: A new author was added in CDDIS-22-2707RR

发起会议
2022-09-28 19:41:16

发件人： "Honghe Zhang" <honghezhang@zju.edu.cn>

收件人： pyliu@zju.edu.cn

抄 送： 21518450@zju.edu.cn 21718673@zju.edu.cn 3110102477@zju.edu.cn 389088167@qq.com 3180102403@zju.edu.cn .. [还有7个联系人]

Hi Pengyuan,

I agree.

Best,

Honghe Zhang

———原始邮件———
发件人:pyliu@zju.edu.cn
发送时间:2022-09-28 18:00:18 (星期三)
收件人：21518450@zju.edu.cn, 21718673@zju.edu.cn, 3110102477@zju.edu.cn, 389088167@qq.com, 3180102403@zju.edu.cn, jial_jacky@163.com, lbj@zju.edu.cn, phoebe837@163.com, 3195024@zju.edu.cn, 3197061@zju.edu.cn, honghezhang@zju.edu.cn, "陆燕" <yanlu76@zju.edu.cn>, "刘鹏渊" <pyliu@zju.edu.cn>, renjy1998@zju.edu.cn
抄送：
主题：RE: A new author was added in CDDIS-22-2707RR

Dear All:

Jiayi Ren has been added to the author list for her contribution to data analysis in a revised manuscript submitted to *Cell Death & Disease* entitled "LCAT1 is an oncogenic LncRNA by stabilizing IGF2BP2-CDC6 axis". If you agree to this change, please reply to this email "I agree" and enter your name.

Thanks for your contribution to this paper!

Pengyuan Liu

Re: RE: A new author was added in CDDIS-22-2707RR

2022-09-29 12:00:32

发件人：yanlu76@zju.edu.cn

收件人：pyliu@zju.edu.cn

抄 送：21518450@zju.edu.cn 21718673@zju.edu.cn 3110102477@zju.edu.cn 389088167@qq.com 3180102403@zju.edu.cn .. [↓还有7个联系人]

Hi Pengyuan,

I agree to add her as co-author for her contribution in the revision.

Best,

Yan

——原始邮件——
发件人:pyliu@zju.edu.cn
发送时间:2022-09-28 18:00:18 (星期三)
收件人: 21518450@zju.edu.cn, 21718673@zju.edu.cn, 3110102477@zju.edu.cn, 389088167@qq.com, 3180102403@zju.edu.cn, jial_jacky@163.com, lbj@zju.edu.cn, phoebe837@163.com, 3195024@zju.edu.cn, 3197061@zju.edu.cn, honghezhang@zju.edu.cn, "陆燕" <yanlu76@zju.edu.cn>, "刘鹏渊" <pyliu@zju.edu.cn>, renjy1998@zju.edu.cn
抄送:
主题: RE: A new author was added in CDDIS-22-2707RR

Dear All:

Jiayi Ren has been added to the author list for her contribution to data analysis in a revised manuscript submitted to *Cell Death & Disease* entitled "LCAT1 is an oncogenic LncRNA by stabilizing IGF2BP2-CDC6 axis". If you agree to this change, please reply to this email "I agree" and enter your name.

Thanks for your contribution to this paper!

Pengyuan Liu

Re: RE: A new author was added in CDDIS-22-2707RR

2022-09-28 18:02:26

发件人：pyliu@zju.edu.cn

收件人："刘鹏渊" <pyliu@zju.edu.cn>

I agree

Pengyuan Liu

——原始邮件——
发件人:pyliu@zju.edu.cn
发送时间:2022-09-28 18:00:18 (星期三)
收件人: 21518450@zju.edu.cn, 21718673@zju.edu.cn, 3110102477@zju.edu.cn, 389088167@qq.com, 3180102403@zju.edu.cn, jial_jacky@163.com, lbj@zju.edu.cn, phoebe837@163.com, 3195024@zju.edu.cn, 3197061@zju.edu.cn, honghezhang@zju.edu.cn, "陆燕" <yanlu76@zju.edu.cn>, "刘鹏渊" <pyliu@zju.edu.cn>, renjy1998@zju.edu.cn
抄送:
主题: RE: A new author was added in CDDIS-22-2707RR

Dear All:

Jiayi Ren has been added to the author list for her contribution to data analysis in a revised manuscript submitted to *Cell Death & Disease* entitled "LCAT1 is an oncogenic LncRNA by stabilizing IGF2BP2-CDC6 axis". If you agree to this change, please reply to this email "I agree" and enter your name.

Thanks for your contribution to this paper!

Pengyuan Liu
